# Supplementary material for: LiteFeatNet: A parameter-efficient and performance-centric deep learning model for multi-ocular disease identification using intermediate feature reduction from fundus images
Source: PLoS One. 2026 May 11;21(5):e0347867. doi: 10.1371/journal.pone.0347867 (PMC13160359; doi:10.1371/journal.pone.0347867)
Supplement: Attachment — Submitted filename: Response to Reviewers.docx [file pone.0347867.s001.docx]

**27-10-2025**

**Academic Editor,**

**PLOS ONE.**

**Subject: Response to reviewer comments for manuscript PONE-D-25-44797**

**Respected Editor,**

First of all, we would like to thank your good self and the reviewers for your valuable time and insightful comments on our manuscript. We have carefully revised the paper according to the comments, suggestions, requirements, and guidelines communicated via decision letter. The suggested revisions have improved the quality and clarity of the manuscript.

Below is a detailed, point-by-point response to each requirement and comment. The comments and responses are shown in tabular format. All changes made in the revised manuscript are highlighted in red color.

**Journal Requirements:**

| **Requirement** | **Response** |
| --- | --- |
| **PLOS ONE’s style requirements** | We sincerely thank the editorial team for this guidance and providing us the useful resources to style our manuscript according to the journal’s requirements. The manuscript (including author affiliations, figures, and tables) has been carefully revised to fully comply with the PLOS ONE style templates. Moreover, the naming conventions specified in the email and PLOS ONE style templates have been thoroughly reviewed and appropriately applied throughout the submission. |
| **Availability of author generated code without any restrictions** | We appreciate the editor’s observation. The complete pipeline code developed and used for this study has been made publicly available on the GitHub repository without any restrictions. Open source license has also been clearly stated. The hyperlink to this repository is provided in the Data Availability Statement section of the manuscript. |
| **Removal of funding related text from the manuscript** | We thank the editorial team for this guideline. According to the guidelines, all funding-related information has been carefully removed from the Acknowledgment section and other parts of the manuscript. |
| **Upload minimal dataset on recommended repositories** | We appreciate the editor’s valuable guidance. A comprehensive Data Availability Statement has been added to the manuscript, and hyperlinks to the recommended repositories of the dataset(s) used in this study have been provided within the statement. |
| **Reviewers recommendation to cite specific articles** | We respectfully note that no specific articles were recommended by the reviewers for citation. Therefore, no additional citations have been added in this regard. |

**Additional Editor Comments:**

| **Requirement** | **Response** |
| --- | --- |
| **Make code used in the study publicly available via trusted platform** | We thank the editor for this important suggestion to meet journal’s standard for transparency, scientific rigor and clarity. The complete pipeline code used for this study has been made publicly available on the GitHub platform without any restrictions. The corresponding access link has been provided in the Data Availability Statement section of the manuscript. |
| **Improve quality of English language** | We sincerely thank the editor for this valuable suggestion. The entire manuscript has been carefully reviewed, and necessary corrections have been made to improve the clarity, coherence, and overall language quality. |
| **Incorporate recent and relevant studies from the past few years** | We appreciate the editor’s valuable feedback to contextualize the research study. The Literature Review section has been updated to incorporate recent and relevant studies published in the past few years, thereby strengthening the context and foundation of the study. |
| **Prepare detailed response letter addressing each of reviewer’s comments** | We thank the editor for providing the guidance necessary to respond to points raised by the reviewers. The response letter has been carefully prepared in accordance with the instructions provided in the decision letter for major revision. |

**Reviewer 1 Comments:**

We respectfully note that Reviewer 1 did not raise any specific points or questions. No changes or article recommendations were suggested by Reviewer 1 for implementation or citation.

**Reviewer 2 Comments:**

We respectfully acknowledge that Reviewer 2 did not provide any specific comments or queries. No revisions or citation suggestions were proposed by Reviewer 2 for inclusion in the manuscript.

**Summary of Major Changes**

1. Manuscript style is made in accordance with PLOS ONE’s style requirements. Naming conventions are updated, as advised. Funding related information has also been removed from the manuscript.
2. Data Availability Statement has been added in the manuscript. Hyperlinks of recommended repositories hosting the dataset(s) have been added in the Data Availability Statement. Moreover, the code used in this study is uploaded on the GitHub platform and made available to the public without any restrictions. Access link has been provided in the Data Availability Statement.
3. Literature review is updated and some recent studies have been incorporated. Moreover, all the recent studies are thoroughly analyzed for relevance to our study.
4. Manuscript has been thoroughly checked for spelling mistakes, grammatical errors and other sentence structure. Changes have been made to improve the quality of English language.

We sincerely thank the reviewers and the editorial team for their constructive input. We hope the revised version meets the journal’s standards for publication.

Sincerely,

Usman Rafi

(On behalf of all authors)
Department of Computer Science,

University of Agriculture, Faisalabad, Punjab, Pakistan
usmanrafiuafcs@gmail.com
