# Supplementary material for: LiteFeatNet: A parameter-efficient and performance-centric deep learning model for multi-ocular disease identification using intermediate feature reduction from fundus images
Source: PLoS One. 2026 May 11;21(5):e0347867. doi: 10.1371/journal.pone.0347867 (PMC13160359; doi:10.1371/journal.pone.0347867)
Supplement: Attachment — Submitted filename: Response_to_Reviewers_auresp_2.docx [file pone.0347867.s002.docx]

**19-03-2026**

**Academic Editor,**

**PLOS ONE.**

**Subject: Response to reviewer’s comments for the manuscript PONE-D-25-44797R1**

**Respected Editor,**

First of all, we would like to thank the esteemed editor and the reviewers for their valuable time, insightful comments, and suggestions on the submitted manuscript (PONE-D-25-44797R1). Secondly, we are also grateful to the editor and reviewers for their encouraging comments and for recognizing the merits of our work.

We have carefully revised our manuscript in response to the concerns, comments, suggestions, requirements, and guidelines raised by the reviewers and communicated via decision letter. The suggested revisions have improved the quality and clarity of the manuscript.

Below is a detailed, point-by-point response to each requirement and comment in accordance with various sections and concerns, as given in the decision letter. The comments and responses are presented in tabular format, in accordance with the decision letter. All changes made in the revised manuscript are mentioned in this response letter using highlighted text (italic fonts in red color). The locations, where amendments are made, are also highlighted in this response letter (bold + underlined fonts in red color) for the sake of convenience. Revised manuscript (both with and without track changes) are included in this response to revision for our manuscript. We hope that this revised version of manuscripts meets the journal’s standards for publication and effectively addresses all the concerns raised.

**Journal Requirements:**

| **Requirement** | **Response** |
| --- | --- |
| If the reviewer comments include a recommendation to cite specific previously published works, please review and evaluate these publications to determine whether they are relevant and should be cited. There is no requirement to cite these works unless the editor has indicated otherwise | We would like to cordially thank the esteemed editor for providing us with guidelines. We carefully reviewed all the comments from the reviewers. No specific recommendations for citing previously published works were made by the reviewers. As a result, no additional references / citations were added in this revised manuscript upon recommendations. |
| Please review your reference list to ensure that it is complete and correct. If you have cited papers that have been retracted, please include the rationale for doing so in the manuscript text, or remove these references and replace them with relevant current references. Any changes to the reference list should be mentioned in the rebuttal letter that accompanies your revised manuscript. If you need to cite a retracted article, indicate the article’s retracted status in the References list and also include a citation and full reference for the retraction notice. | We thank the editor for highlighting this important point. We have carefully reviewed the entire reference list to ensure that all citations are complete, accurate, and up to date. None of the cited articles have been retracted. Therefore, no references required removal or replacement in this regard.  However, while addressing reviewer’s comments during the revision process, several new references were incorporated in the manuscript to contextualize and strengthen our work.  We have also cross-checked all the references and to ensure completeness and consistency in formatting according to the journal’s guidelines. Therefore, we have updated the **References** section as follows:  **References**  **Line # 1282-1522**  *1. Burton MJ, Ramke J, Marques AP, Bourne RRA, Congdon N, Jones I, et al. The Lancet Global Health Commission on Global Eye Health: vision beyond 2020. Lancet Glob Heal [Internet]. 2021;9:e489–551. Available from: https://www.sciencedirect.com/science/article/pii/S2214109X20304885*  *2. Grzybowski A, Jin K, Zhou J, Pan X, Wang M, Ye J, et al. Retina Fundus Photograph-Based Artificial Intelligence Algorithms in Medicine: A Systematic Review. Ophthalmol Ther [Internet]. 2024;13:2125–49. Available from: https://doi.org/10.1007/s40123-024-00981-4*  *3. Saqib SM, Asghar MZ, Iqbal M, Al-Rasheed A, Khan MA, Ghadi Y, et al. DenseHillNet: a lightweight CNN for accurate classification of natural images. PeerJ Comput Sci [Internet]. 2024;10:1–21. Available from: https://peerj.com/articles/cs-1995/*  *4. L V, Ravikumar A, Sriraman H. Diabetic Retinopathy Detection Using Smartphone Fundoscopy: Advancing Healthcare Equity through Intelligent Edge Computing. Procedia Comput Sci [Internet]. 2025. p. 2190–9. Available from: https://www.sciencedirect.com/science/article/pii/S1877050925015728*  *5. Romero R, Celard P, Sorribes-Fdez JM, Seara Vieira A, Iglesias EL, Borrajo L. MobyDeep: A lightweight CNN architecture to configure models for text classification. Knowledge-Based Syst [Internet]. 2022;257:109914. Available from: https://www.sciencedirect.com/science/article/pii/S0950705122010073*  *6. Lai N, Dewi DA, Maidin SS, Xiao W, Zhao S, Hu Q. A comprehensive review of lightweight deep learning models for edge computing with future directions. Discov Comput [Internet]. 2026;29:110. Available from: https://doi.org/10.1007/s10791-026-10021-3*  *7. Vohra H, Hasan MK, Norul Huda Sheikh Abdullah S, Islam S, Hadi Abd Rahman A, Bhojake SN, et al. A Low-Cost AI-Powered System for Early Detection of Diabetic Retinopathy and Ocular Diseases in Resource Limited Settings. IEEE Access. 2025;13:97322–36.*  *8. Hassan B, Ahmed R, Li B, Noor A, Hassan Z ul. A comprehensive study capturing vision loss burden in Pakistan (1990-2025): Findings from the Global Burden of Disease (GBD) 2017 study. PLoS One [Internet]. 2019;14:1–19. Available from: https://doi.org/10.1371/journal.pone.0216492*  *9. Benzinger L, Ursin F, Balke W-T, Kacprowski T, Salloch S. Should Artificial Intelligence be used to support clinical ethical decision-making? A systematic review of reasons. BMC Med Ethics [Internet]. 2023;24:48. Available from: https://doi.org/10.1186/s12910-023-00929-6*  *10. Jabbar MK, Yan J, Xu H, Rehman ZU, Jabbar A. Transfer Learning-Based Model for Diabetic Retinopathy Diagnosis Using Retinal Images. Brain Sci. 2022;12.*  *11. Jabbar A, Naseem S, Li J, Mahmood T, Jabbar MK, Rehman A, et al. Deep Transfer Learning-Based Automated Diabetic Retinopathy Detection Using Retinal Fundus Images in Remote Areas. Int J Comput Intell Syst [Internet]. 2024;17:135. Available from: https://doi.org/10.1007/s44196-024-00520-w*  *12. Shakya M, Patel R, Joshi S. A comprehensive analysis of deep learning and transfer learning techniques for skin cancer classification. Sci Rep [Internet]. 2025;15:4633. Available from: https://doi.org/10.1038/s41598-024-82241-w*  *13. Fu Y, Guo L, Huang F. A lightweight CNN model for pepper leaf disease recognition in a human palm background. Heliyon [Internet]. 2024;10. Available from: https://doi.org/10.1016/j.heliyon.2024.e33447*  *14. Prince RH, Mamun A Al, Peyal HI, Miraz S, Nahiduzzaman M, Khandakar A, et al. CSXAI: a lightweight 2D CNN-SVM model for detection and classification of various crop diseases with explainable AI visualization. Front Plant Sci [Internet]. 2024;Volume 15. Available from: https://www.frontiersin.org/journals/plant-science/articles/10.3389/fpls.2024.1412988*  *15. Ghislain F, Beaudelaire ST, Atangana R, Daniel T. An improved deep learning approach for automated detection of multiclass eye diseases. Array [Internet]. 2025;27:100452. Available from: https://www.sciencedirect.com/science/article/pii/S2590005625000797*  *16. Qasim DK, Jebur SA, Ali LR, Khalaf AJM, Hussain AJ. Lightweight Convolutional Neural Networks for Retinal Disease Classification [Internet]. 2025. Available from: https://arxiv.org/abs/2506.03186*  *17. Ejaz S, Baig R, Ashraf Z, Alnfiai MM, Alnahari MM, Alotaibi RM. A deep learning framework for the early detection of multi-retinal diseases. PLoS One [Internet]. 2024;19:1–23. Available from: https://doi.org/10.1371/journal.pone.0307317*  *18. Ejaz S, Zia HU, Majeed F, Shafique U, Altamiranda SC, Lipari V, et al. Fundus image classification using feature concatenation for early diagnosis of retinal disease. Digit Heal [Internet]. 2025;11:20552076251328120. Available from: https://doi.org/10.1177/20552076251328120*  *19. Nawaz A, Ali T, Mustafa G, Babar M, Qureshi B. Multi-Class Retinal Diseases Detection Using Deep CNN With Minimal Memory Consumption. IEEE Access. 2023;11:56170–80.*  *20. Chavan R, Pete D. Automatic multi-disease classification on retinal images using multilevel glowworm swarm convolutional neural network. J Eng Appl Sci [Internet]. 2024;71:26. Available from: https://doi.org/10.1186/s44147-023-00335-0*  *21. Aqsa Aslam Saima Farhan MAKFAAAFK. Convolutional Neural Network-Based Classification of Multiple Retinal Diseases Using Fundus Images. Intell Autom \& Soft Comput [Internet]. 2023;36:2607–22. Available from: http://www.techscience.com/iasc/v36n3/51900*  *22. Chowa SS, Bhuiyan MRI, Payel IJ, Karim A, Khan IU, Montaha S, et al. A Low Complexity Efficient Deep Learning Model for Automated Retinal Disease Diagnosis. J Healthc Informatics Res [Internet]. 2025;9:1–40. Available from: https://doi.org/10.1007/s41666-024-00182-5*  *23. Elkholy M, Marzouk MA. Deep learning-based classification of eye diseases using Convolutional Neural Network for OCT images. Front Comput Sci [Internet]. 2024;Volume 5-. Available from: https://www.frontiersin.org/journals/computer-science/articles/10.3389/fcomp.2023.1252295*  *24. Kher G, Mehra S, Bala R, Singh RP. DeB5-XNet: An explainable ensemble model for ocular disease classification using feature extraction and Grad-CAM. Informatics Med Unlocked [Internet]. 2025;54:101632. Available from: https://www.sciencedirect.com/science/article/pii/S2352914825000206*  *25. Das PK, Pumrin S. Diabetic retinopathy classification: performance evaluation of pre-trained lightweight CNN using imbalance dataset. Eng J. 2024;28:13–25.*  *26. Rimaru D, Nehme A, Alhussein M, Mahbub K, Aurangzeb K, Khan A. A Mobile Deep Learning Classification Model for Diabetic Retinopathy. Elektron ir Elektrotechnika [Internet]. 2024;30:45–52. Available from: https://eejournal.ktu.lt/index.php/elt/article/view/38674*  *27. Choudhary A, Ahlawat S, Urooj S, Pathak N, Lay-Ekuakille A, Sharma N. A Deep Learning-Based Framework for Retinal Disease Classification. Healthcare [Internet]. 2023;11. Available from: https://www.mdpi.com/2227-9032/11/2/212*  *28. Jaimes WJ, Arenas WJ, Navarro HJ, Altuve M. Detection of retinal diseases from OCT images using a VGG16 and transfer learning. Discov Appl Sci [Internet]. 2025;7:160. Available from: https://doi.org/10.1007/s42452-025-06565-6*  *29. Hasan MN, Pial MER, Das S, Siddique N, Wang H. DIA-VXNET: A framework for automated diabetic eye disease detection using transfer learning with feature fusion network. Biomed Signal Process Control [Internet]. 2025;100:106907. Available from: https://www.sciencedirect.com/science/article/pii/S1746809424009650*  *30. Flores N, La Rosa J, Tuesta S, Izquierdo L, Henriquez M, Mauricio D. Hybrid Deep Learning Model for Improved Glaucoma Diagnostic Accuracy. Information [Internet]. 2025;16. Available from: https://www.mdpi.com/2078-2489/16/7/593*  *31. Diaz-Pinto A, Morales S, Naranjo V, Köhler T, Mossi JM, Navea A. CNNs for automatic glaucoma assessment using fundus images: an extensive validation. Biomed Eng Online [Internet]. 2019;18:29. Available from: https://doi.org/10.1186/s12938-019-0649-y*  *32. Devalla SK, Chin KS, Mari J-M, Tun TA, Strouthidis NG, Aung T, et al. A Deep Learning Approach to Digitally Stain Optical Coherence Tomography Images of the Optic Nerve Head. Invest Ophthalmol Vis Sci [Internet]. 2018;59:63–74. Available from: https://doi.org/10.1167/iovs.17-22617*  *33. Zafar A, Kim KS, Ali MU, Byun JH, Kim S-H. A lightweight multi-deep learning framework for accurate diabetic retinopathy detection and multi-level severity identification. Front Med [Internet]. 2025;Volume 12. Available from: https://www.frontiersin.org/journals/medicine/articles/10.3389/fmed.2025.1551315*  *34. Naveen K V, Anoop BN, Siju KS, Kar MK, Venugopal V. EffNet-SVM: A Hybrid Model for Diabetic Retinopathy Classification Using Retinal Fundus Images. IEEE Access. 2025;13:79793–804.*  *35. Sushith M, Sathiya A, Kalaipoonguzhali V, Sathya V. A hybrid deep learning framework for early detection of diabetic retinopathy using retinal fundus images. Sci Rep [Internet]. 2025;15:15166. Available from: https://doi.org/10.1038/s41598-025-99309-w*  *36. Arnob AKB, Chayon MHR, Al Farid F, Husen MN, Ahmed F. A Lightweight CNN for Multiclass Retinal Disease Screening with Explainable AI. J Imaging [Internet]. 2025;11. Available from: https://www.mdpi.com/2313-433X/11/8/275*  *37. Mowla N, Mowla MN, Rabie K, Alsinglawi B. A Lightweight Deep Learning Model for Retinopathy of Prematurity Classification in eHealth Applications. 2025 Int Wirel Commun Mob Comput. 2025. p. 227–32.*  *38. Pachade S, Porwal P, Thulkar D, Kokare M, Deshmukh G, Sahasrabuddhe V, et al. Retinal Fundus Multi-Disease Image Dataset (RFMiD): A Dataset for Multi-Disease Detection Research. Data [Internet]. 2021;6. Available from: https://www.mdpi.com/2306-5729/6/2/14*  *39. Pachade S, Porwal P, Thulkar D, Kokare M, Deshmukh G, Sahasrabuddhe V, et al. Retinal Disease Classification [Internet]. Retin. Fundus Multi-Disease Image Dataset A Dataset Multi-Disease Detect. Res. 2021 [cited 2025 Jul 2]. Available from: https://www.kaggle.com/datasets/andrewmvd/retinal-disease-classification*  *40. Pachade S, Porwal P, Thulkar D, Kokare M, Deshmukh G, Sahasrabuddhe V, et al. Retinal Fundus Multi-disease Image Dataset (RFMiD) [Internet]. IEEE Dataport; 2020 [cited 2025 Jul 2]. Available from: https://dx.doi.org/10.21227/s3g7-st65*  *41. Pachade S, Porwal P, Kokare M, Deshmukh G, Sahasrabuddhe V, Luo Z, et al. RFMiD: Retinal Image Analysis for multi-Disease Detection challenge. Med Image Anal [Internet]. 2025;99:103365. Available from: https://www.sciencedirect.com/science/article/pii/S1361841524002901*  *42. Mayya V, S SK, Kulkarni U, Surya DK, Acharya UR. An empirical study of preprocessing techniques with convolutional neural networks for accurate detection of chronic ocular diseases using fundus images. Appl Intell [Internet]. 2023;53:1548–66. Available from: https://doi.org/10.1007/s10489-022-03490-8*  *43. Jha R, Bhattacharjee V, Mustafi A. Transfer Learning with Feature Extraction Modules for Improved Classifier Performance on Medical Image Data. Sci Program [Internet]. 2022;2022:4983174. Available from: https://onlinelibrary.wiley.com/doi/abs/10.1155/2022/4983174*  *44. Jia W, Sun M, Lian J, Hou S. Feature dimensionality reduction: a review. Complex Intell Syst [Internet]. 2022;8:2663–93. Available from: https://doi.org/10.1007/s40747-021-00637-x*  *45. Solmaz ÖA, Tasci B. ViSwNeXtNet Deep Patch-Wise Ensemble of Vision Transformers and ConvNeXt for Robust Binary Histopathology Classification. Diagnostics [Internet]. 2025;15. Available from: https://www.mdpi.com/2075-4418/15/12/1507*  *46. Ahmed MR, Shehata MS, Lasserre P. Integrating lightweight convolutional neural network with entropy-informed channel attention and adaptive spatial attention for OCT-based retinal disease classification. Comput Biol Med [Internet]. 2025;190:110071. Available from: https://www.sciencedirect.com/science/article/pii/S0010482525004226*  *47. Islam S, Deo RC, Barua PD, Soar J, Acharya UR. Novel Deep Learning Model for Glaucoma Detection Using Fusion of Fundus and Optical Coherence Tomography Images. Sensors [Internet]. 2025;25. Available from: https://www.mdpi.com/1424-8220/25/14/4337*  *48. Maçin G, Genç F, Taşcı B, Dogan S, Tuncer T. KidneyNeXt: A Lightweight Convolutional Neural Network for Multi-Class Renal Tumor Classification in Computed Tomography Imaging. J Clin Med [Internet]. 2025;14. Available from: https://www.mdpi.com/2077-0383/14/14/4929*  *49. Reyad M, Sarhan AM, Arafa M. A modified Adam algorithm for deep neural network optimization. Neural Comput Appl [Internet]. 2023;35:17095–112. Available from: https://doi.org/10.1007/s00521-023-08568-z*  *50. Mahbod A, Saeidi N, Hatamikia S, Woitek R. Evaluating pre-trained convolutional neural networks and foundation models as feature extractors for content-based medical image retrieval. Eng Appl Artif Intell [Internet]. 2025;150:110571. Available from: https://www.sciencedirect.com/science/article/pii/S0952197625005718*  *51. De Diego IM, Redondo AR, Fernández RR, Navarro J, Moguerza JM. General Performance Score for classification problems. Appl Intell [Internet]. 2022;52:12049–63. Available from: https://doi.org/10.1007/s10489-021-03041-7*  *52. Nettur SB, Karpurapu S, Nettur U, Gajja LS, Myneni S, Dusi A, et al. UltraLightSqueezeNet: A Deep Learning Architecture for Malaria Classification With Up to 54× Fewer Trainable Parameters for Resource Constrained Devices. IEEE Access. 2025;13:89428–40.*  *53. G P, Walikar A, Ahammed SR, Suresh Y, Parihar H, Manoj V. Deep Learning Applications in Embedded Systems and IoT Devices. 2025 13th Int Conf Smart Grid. 2025. p. 518–26.*  *54. Baller SP, Jindal A, Chadha M, Gerndt M. DeepEdgeBench: Benchmarking Deep Neural Networks on Edge Devices. 2021 IEEE Int Conf Cloud Eng. 2021. p. 20–30.*  *55. Martinez-Alpiste I, Golcarenarenji G, Wang Q, Alcaraz-Calero JM. Smartphone-based real-time object recognition architecture for portable and constrained systems. J Real-Time Image Process [Internet]. 2022;19:103–15. Available from: https://doi.org/10.1007/s11554-021-01164-1*  *56. Vincent, Darian G, Surantha N. Performance Evaluation of Convolutional Neural Network (CNN) for Skin Cancer Detection on Edge Computing Devices. Appl Sci [Internet]. 2025;15. Available from: https://www.mdpi.com/2076-3417/15/6/3077*  *57. Ghislain F, Beaudelaire ST, Atangana R, Daniel T. Real-time deep learning for multi-label retinal disease diagnosis with embedded system. Comput Struct Biotechnol Reports [Internet]. 2025;2:100035. Available from: https://www.sciencedirect.com/science/article/pii/S2950363925000067*  *58. Zhuang W, Hascoet T, Chen X, Takashima R, Takiguchi T, Ariki Y. Convolutional Neural Networks Inference Memory Optimization with Receptive Field-Based Input Tiling. APSIPA Trans Signal Inf Process [Internet]. 2023;12:1–20. Available from: https://doi.org/10.1561/116.00000015*  *59. Demir B, Domazet E, Mechkaroska D. Overview of Memory-Efficient Architectures for Deep Learning in Real-Time Systems. Eng Proc [Internet]. 2025;104. Available from: https://www.mdpi.com/2673-4591/104/1/77*  *60. Mittal P. A comprehensive survey of deep learning-based lightweight object detection models for edge devices. Artif Intell Rev [Internet]. 2024;57:242. Available from: https://doi.org/10.1007/s10462-024-10877-1*  *61. Panchal S, Naik A, Kokare M, Pachade S, Naigaonkar R, Phadnis P, et al. Retinal Fundus Multi-Disease Image Dataset (RFMiD) 2.0: A Dataset of Frequently and Rarely Identified Diseases. Data [Internet]. 2023;8. Available from: https://www.mdpi.com/2306-5729/8/2/29*  *62. Panchal S, Naik A, Kokare M, Pachade S, Phadnis P, Bhange A. Retinal Fundus Multi-Disease Image Dataset (RFMiD) 2.0 [Internet]. 2023 [cited 2025 Jul 2]. Available from: https://zenodo.org/records/7505822*  *63. Sharmin S, Rashid MR, Khatun T, Hasan MZ, Uddin MS, Marzia. A dataset of color fundus images for the detection and classification of eye diseases. Data Br [Internet]. 2024;57:110979. Available from: https://www.sciencedirect.com/science/article/pii/S2352340924009417*  *64. Rashid MR, Sharmin S, Khatun T, Hasan MZ, Uddin MS. Eye Disease Image Dataset [Internet]. A dataset Color fundus images Detect. Classif. eye Dis. 2024 [cited 2026 Feb 20]. Available from: https://data.mendeley.com/datasets/s9bfhswzjb/1*  *65. Rashid MR, Sharmin S, Khatun T, Hasan MZ, Uddin MS. Eye Disease Image Dataset - Mendeley [Internet]. A dataset Color fundus images Detect. Classif. eye Dis. 2024 [cited 2026 Feb 20]. Available from: https://www.kaggle.com/datasets/linabennaa/eye-disease-image-dataset-mendeley*  *66. Agarwal D, Bhargava A, Alsharif MH, Saeed S, Alsharif NHM. Automatic diagnosis of age-related macular degeneration using machine learning and image processing techniques. Sci Rep [Internet]. 2026;16:5037. Available from: https://doi.org/10.1038/s41598-026-35428-2* |

**Additional Editor Comments:**

We sincerely thank the esteemed editor for positive evaluation of our manuscript, recognition of its merit, and potential contribution in the research domain. We are also grateful for the constructive feedback provided. We have carefully revised our manuscript by carefully addressing all the comments and suggestions communicated through decision letter.

**Reviewer # 2 Comments:**

We sincerely thank reviewer for the positive evaluation, encouraging feedback, and kind remarks on our manuscript. We are also pleased and appreciate the reviewer’s recognition of the quality of our work.

**Reviewer # 3 Comments:**

We thank and appreciate the reviewer for the careful evaluation of our manuscript and for insightful feedback, comments, and suggestions. These remarks have helped us improve the clarity and quality of our manuscript. Our point-by-point response is given below:

**Major Concerns:**

**# 1**

The authors present DIFE and PFMR as novel contributions. However, these techniques are not new in themselves. Extracting intermediate features from CNNs is a well-established practice in transfer learning, and PFMR is essentially a 1×1 convolution for channel reduction, a foundational technique dating back to Network-in-Network (2014) and used extensively in Inception, ResNet bottlenecks, and MobileNet architectures. The manuscript should more transparently acknowledge that the contribution lies in the specific combination and configuration of these existing techniques for retinal disease classification, rather than presenting them as methodological innovations.

**Response**

We are extremely grateful to the reviewer for highlighting this important concern regarding the DIFE and PFMR architectural components. We also fully agree that the components integrated in our proposed architecture, such as intermediate feature extraction and 1x1 convolution layer, are well-established techniques in deep learning, as used in Inception, ResNet bottlenecks, and MobileNet. However, our intention was not to claim novelty in these operations. Rather, these components were deliberatively integrated to reduce computational complexity, inference time, and model parameters while preserving discriminative feature representations.

Specifically, in DIFE, feature maps are extracted from the NASNetMobile backbone prior to the ReLU activation layer. This strategy allowed the model to utilize feature-rich intermediate representations (7x7x1056) while avoiding additional computation by ReLU. In PFMR, a 1x1 convolutional operation reduces the channel-dimension of DIFE-extracted features to 7x7x512 and a ReLU activation is performed to the reduced feature maps. This further lowers the computational burden of subsequent operations in the classification module.

To avoid any ambiguity, we have carefully revised our manuscript. We have clarified that the actual contribution of this work lies in task-oriented integration and configuration of these operations within a unified architecture for retinal disease classification, rather than presenting them as methodological innovations. Therefore, amendments made are as follows:

**Abstract**

**Line # 20-26**

*To enhance robustness and reduce computational time, a pre-trained NASNetMobile backbone is employed, and a method for discriminative feature extraction from deep intermediate layers is proposed. The extracted features are refined using a spatially-aware feature map reduction module and classified using a custom classification module with fewer number of trainable parameters, reduced computational resource requirements, and computational time.*

**1 Introduction**

**Line # 108-114**

*In this study, a performance-centric, computationally efficient, and robust deep learning model is proposed that leverages transfer learning for accurate diagnosis of multiple retinal conditions. Unlike conventional transfer learning setups, intermediate-level disease-rich discriminative features were extracted from the backbone using Deep Intermediate Feature Extraction (DIFE), refined using a Pointwise Feature Map Reduction (PFMR) module, and fed to a Lightweight Custom Classification (LCC) module for predictions.*

**1.1 Research contributions**

**Line # 132-135**

2) *Demonstrate that DIFE-based intermediate features, when processed through the PFMR module, achieve superior performance and reduce computational resource requirements in comparison to full-layered feature extraction from the pre-trained CNNs.*

**3.4 Deep Intermediate Feature Extraction (DIFE)**

**Line # 403-416**

*The final feature-extraction layer of NASNetMobile generates an activated feature map of 7x7x1056 resulting from deep concatenated features. In a study, [16] extracted 7x7x1056 Rectified Linear Unit (ReLU)-activated features from the final layer of the feature extractor.*

*However, to make the training process time-efficient and the classifier more robust, the NASNetMobile backbone architecture was carefully analysed. Our approach extracts deep intermediate features from the backbone without losing spatial or channel information, and fully leverages the refined features extracted by the backbone. As a result, deep intermediate features with a spatial dimension of 7x7x1056 were extracted prior to the ReLU activation from the* ***normal_cat_12 layer*** *of the NASNetMobile backbone, ensuring that the features were sufficiently deep to capture all relevant pathological patterns properly while reducing computational time. Fig 4 shows the extraction mechanism of the proposed DIFE.*

***Fig 4.*** ***DIFE from the backbone architecture.***

**3.5 Point-wise Feature Map Reduction (PFMR) module**

**Line # 429-442**

*To achieve parameter reduction and computational efficiency, this study integrates the PFMR module for spatial-aware dimension reduction and feature refinement. The PFMR module, as shown in Fig 5, performs two operations on the input feature maps.*

***Fig 5. PFMR module.***

*First, it employs a point-wise 1x1 convolutional layer that outputs refined feature maps with 512 channels, with spatial dimensions identical to those of the DIFE-extracted features. The convolutional layer refines the input features by removing redundant and low-information feature maps, thereby producing a set of feature maps with the most relevant and important discriminative features. Secondly, the output feature maps are ReLU-activated in an efficient manner. The PFMR module preserves the spatial semantic richness of backbone-extracted features while reducing the model’s parameter count, making it lighter and faster. Algorithm 1 defines the PFMR’s action mechanism for dimension reduction of the input feature maps.*

**4.1 Comparison of parameters and model size**

**Line # 628-631**

*Hence, the parameter trade-off achieved with the PFMR and LCC modules reduced the number and size of parameters sufficiently, making it suitable for memory-efficient and faster computations on resource-constrained across numerous computing devices, while remaining complex enough to learn inherent label-specific patterns.*

**4.4 Overall performance metrics**

**Line # 777-783**

*The results verify that the LiteFeatNet possesses superior capability to learn disease-wise discriminative features. Moreover, incorporating DIFE from the backbone, an optimized feature refinement strategy (PFMR), and an LCC module enabled the model to achieve robust performance for classifying NL, DR, and MH, with reduced computational cost, particularly the inference time (1.32 seconds), making it a suitable choice for deployment in resource-scarce environments, particularly with a higher patient counts.*

**5.5 Evaluation metrics**

**Line # 975-978**

*Hence, the LiteFeatNet demonstrated robust generalization on the RFMiD 2.0, offering a superior trade-off between performance and efficiency thanks to its unique parameter optimization and compact size powered by integrating DIFE, PFMR, and the LCC modules.*

**6 Scalability evaluation**

**Line # 1043-1045**

*In contrast, our proposed architecture captured disease-relevant features effectively and efficiently through the synergistic combination of DIFE, PFMR and LCC modules.*

**Line # 1074-1079**

*The results also validate that the integration of the architectural components (DIFE, PFMR, and LCC) has enabled our proposed framework to be robust, scalable, adaptable, and computationally efficient, beyond the original 3-class experimental scenario, thereby positioning it as a viable automated diagnostic tool suitable for larger multi-class problems in real-world clinical deployment.*

**7 Ablation study**

**Line # 1081-1084**

*To evaluate the individual and combined impact of DIFE and the PFMR module, a comprehensive ablation study is also conducted using the RFMiD. To obtain detailed insights into the integration of DIFE and PFMR, we conducted ablation experiments for our proposed framework and two backbone architectures.*

7.1 Ablation experiments for the proposed framework

Line # 1086-1088

*To understand the impact of integrating DIFE and PFMR in our proposed architecture, we conducted ablation experiments using four different model variants (model title suffixes: A, B, C, and D) of our proposed architecture.*

**Line # 1152-1157**

*Hence, this ablation study strongly validates that both DIFE and PFMR contribute implicitly to achieving the optimal classification performance while maintaining computational efficiency. On the whole, the integration of DIFE and PFMR modules made LiteFeatNet outperformed all state-of-the-art transfer learning methods and well-established architectures across all performance metrics, making it efficient and robust enough to avoid misdiagnosis in healthy patients.*

**7.2 Ablation experiments for other backbone architectures**

**Line # 1226-1229**

*This, once again confirms that the DIFE and PFMR synergy empowers transfer learning models to be computationally efficient, thereby enabling deployment-level efficiency in resource-constrained environments.*

**Line # 1231-1233**

*All results with the configuration utilizing both DIFE and PFMR confirm the synergistic effect of DIFE and PFMR for performance improvement across all the baselines, in particular.*

**8 Conclusions**

**Line # 1241-1244**

*The LiteFeatNet model achieved superior performance compared to numerous standard deep learning architectures due to the incorporation of DIFE, a spatial-loss-aware feature refinement module (PFMR), and a compact 6-layer LCC module.*

**Line # 1251-1253**

*The ablation study also validated the synergistic effect of DIFE and PFMR, identifying it as critical for achieving optimal performance, resource, and computational efficiency from the proposed architecture.*

**# 2**

The manuscript lacks essential reproducibility components. The authors should provide the model implementation code and test scripts, ideally through a public repository. This is particularly important given the claims of superior performance and the specific architectural choices involved in DIFE and PFMR.

**Response**

We appreciate reviewer’s observation regarding the reproducibility component. The complete code has been made publicly available on the GitHub repository. The link to this repository is provided in the **Data Availability Statement** under Code Availability section of the manuscript.

**Data Availability Statement**

**Line # 1278-1280**

***Code availability:*** *The* *code developed for this study is made available on GitHub. The code can be accessed at the following URL:*

[*https://github.com/usmanrafics/LTFTNET*](https://github.com/usmanrafics/LTFTNET)

We hope that this resolves the concern regarding the essential reproducibility components due to the importance of superior performance and the architectural choices involved in DIFE and PFMR.

**# 3**

Training curves are only provided for the proposed LiteFeatNet model. The manuscript does not demonstrate that all comparison models reached training convergence. This is a critical concern, as Table 5 reveals that the EfficientNet models (B0 and V2B0) exhibit pathological behavior, achieving 0% precision and 0% sensitivity for DR and MH classes while predicting NL for all samples. This pattern strongly suggests training failure rather than genuine model limitations. The authors must ensure that all models are correctly and fully trained until convergence before drawing comparative conclusions. The current results for EfficientNet models should either be corrected through proper training or excluded from the comparison with appropriate justification.

**Response**

We are extremely grateful to the reviewer for raising this important concern regarding training convergence for providing a fair comparison as base for analysis. Following the kind suggestion of the reviewer, we have carefully reviewed the accuracy and loss curves for all the models. According to these curves, all the models demonstrated consistent stabilization, thereby confirming that all the models were trained until convergence. For clarity, we have also added these curves (for all the architectures used in this study) and related discussion in **section 4.2** of this revised version of the manuscript. Accuracy curves of all the models are combined into a single figure **Fig. 7** while loss curves are also combined together and presented in a single figure **Fig. 8**. The figure captions are also updated accordingly.

We have also performed a number of additional experiments according to the valuable suggestions of the reviewer and we truly believe that these suggestions have improved the quality of our manuscript greatly. Following these suggestions, we have added an entirely new section **(section 6)** and a sub-section **(section 7.2)** in this revised manuscript in response to **major concern # 4** and **minor concern # 1**, respectively. Therefore, to manage the overall sense, consistency, and length of our manuscript, we have excluded the least performing models from this revised manuscript (including related table entries and corresponding discussion).

The revised manuscript contains results and discussion for the baseline models that demonstrated stable convergence and training. We believe that these amendments significantly strengthens the scientific rigor, methodological validity, and fairness of comparison. The content updates in various sections / sub-sections are as follows:

**4.2 Training performance**

**Line # 636-637**

*The trend analysis of training and validation accuracies for all the models, including LiteFeatNet are illustrated in Fig 7.*

**Line # 640-643**

***Fig 7.*** ***Accuracy plots.***

*(A) CNN-1 [17] (B) DenseNet121 (C) DeB5-XNet [24] (D) DIA-VXNET [29] (E) LightCNN [16] (F) MobileNet (G) MobileNetV2 (H) NAS_m1 [30] (I) NAS_m2 [30] (J) NASNetMobile (K) Proposed Model (LiteFeatNet) (L) Swin V2-Small (M) Swin V2-Tiny*

**Line # 651-656**

*Fig 8 presents the trajectories of the training and validation losses for several models evaluated in this study.*

***Fig 8.*** ***Loss plots.***

*(A) CNN-1 [17] (B) DenseNet121 (C) DeB5-XNet [24] (D) DIA-VXNET [29] (E) LightCNN [16] (F) MobileNet (G) MobileNetV2 (H) NAS_m1 [30] (I) NAS_m2 [30] (J) NASNetMobile (K) Proposed Model (LiteFeatNet) (L) Swin V2-Small (M) Swin V2-Tiny*

**Line # 672-677**

*According to Fig 7, all the architectures also demonstrated a stable learning behavior in terms of accuracy. Moreover, both training and validation accuracies for all evaluated models reach performance plateaus, particularly in later epochs (20 or above). Similarly, the loss curves in Fig 8, provide further confirmation of stable optimization and convergence of all the evaluated architectures. Therefore, both accuracy and loss plots clearly indicated convergence across all the models.*

**Abstract**

**Line # 30-32**

*It outperformed twelve state-of-the-art models, achieving the highest testing accuracy of 90.33%, precision of 90.69%, recall of 90.33%, and F1-score of 90.27%, with a fast, impressive inference time of 4 milliseconds per image.*

**1 Introdcution**

**Line # 116-120**

*Moreover, the proposed model has also outperformed numerous architectures, including 6 cutting-edge transfer learning models: DenseNet121, MobileNet, MobileNetV2, NASNetMobile, Swin Transformer V2-Small, and Swin Transformer V2-Tiny, while achieving faster computation.*

**3.8 Experimental setup**

**Line # 498-502**

*Further, extensive experiments are conducted with numerous architectures LightCNN [16], CNN-1 [17], DeB5-XNet [24], DIA-VXNET [29], NAS_m1 [30], NAS_m2 [30], and six well-established pre-trained models (DenseNet121, MobileNet, MobileNetV2, NASNetMobile, Swin V2-Small and Swin V2-Tiny) due to low computational overhead, particularly for medical image datasets [50].*

**4.1 Comparison of parameters and model size**

**Line # 567**

***Table 3. Comparison of number and size of parameters.***

| ***Model*** | ***Number of Parameters*** | | | ***Size*** | | |
| --- | --- | --- | --- | --- | --- | --- |
|  | ***Total*** | ***Trainable*** | ***Non-Trainable*** | ***Trainable Parameters*** | ***Non-Trainable Parameters*** | ***Entire Model*** |
| *CNN-1 [17]* | *499,011* | *499,011* | *0* | *1.90* | *0.00* | *1.90* |
| *DenseNet121* | *7,040,579* | *3,075* | *7,037,504* | *0.01* | *26.85* | *26.86* |
| *DeB5-XNet [24]* | *37,140,282* | *1,582,083* | *35,558,199* | *6.04* | *135.64* | *141.68* |
| *DIA-VXNET [29]* | *40,923,883* | *5,344,899* | *35,578,984* | *20.39* | *135.72* | *156.11* |
| *LightCNN [16]* | *5,355,159* | *1,085,443* | *4,269,716* | *4.14* | *16.29* | *20.43* |
| *MobileNet* | *4,256,867* | *3,003* | *4,253,864* | *0.01* | *16.23* | *16.24* |
| *MobileNetV2* | *2,261,827* | *3,843* | *2,257,984* | *0.01* | *8.61* | *8.63* |
| *NAS_m1 [30]* | *4,272,887* | *3,171* | *4,269,716* | *0.01* | *16.29* | *16.30* |
| *NAS_m2 [30]* | *17,549,719* | *13,280,003* | *4,269,716* | *50.66* | *16.29* | *66.95* |
| *NASNetMobile* | *4,272,887* | *3,171* | *4,269,716* | *0.01* | *16.29* | *16.30* |
| *Proposed (LiteFeatNet)* | ***5,207,703*** | ***936,963*** | ***4,270,740*** | ***3.57*** | ***16.29*** | ***19.87*** |
| *Swin V2-Small* | *48,970,749* | *2,307* | *48,968,442* | *0.01* | *186.76* | *186.77* |
| *Swin V2-Tiny* | *27,584,877* | *2,307* | *27,582,570* | *0.01* | *105.22* | *105.23* |

**4.2 Training performance**

**Line # 641-643**

*(A) CNN-1 [17] (B) DenseNet121 (C) DeB5-XNet [24] (D) DIA-VXNET [29] (E) LightCNN [16] (F) MobileNet (G) MobileNetV2 (H) NAS_m1 [30] (I) NAS_m2 [30] (J) NASNetMobile (K) Proposed Model (LiteFeatNet) (L) Swin V2-Small (M) Swin V2-Tiny*

**Line # 654-656**

*(A) CNN-1 [17] (B) DenseNet121 (C) DeB5-XNet [24] (D) DIA-VXNET [29] (E) LightCNN [16] (F) MobileNet (G) MobileNetV2 (H) NAS_m1 [30] (I) NAS_m2 [30] (J) NASNetMobile (K) Proposed Model (LiteFeatNet) (L) Swin V2-Small (M) Swin V2-Tiny*

**4.4 Overall performance metrics**

**Line # 694**

***Table 5. Overall performance metrics for RFMiD.***

| ***Model*** | ***Accuracy*** | ***Precision*** | ***Recall*** | ***F1-Score*** | ***Training Time*** | ***Inference Time*** |
| --- | --- | --- | --- | --- | --- | --- |
| *CNN-1 [17]* | *71.27* | *72.41* | *71.27* | *70.89* | *325.65* | *0.91* |
| *DenseNet121* | *89.23* | *89.69* | *89.23* | *89.11* | *416.62* | *1.59* |
| *DeB5-XNet [24]* | *88.67* | *89.19* | *88.67* | *88.61* | *635.35* | *2.96* |
| *DIA-VXNET [29]* | *88.40* | *88.80* | *88.40* | *88.30* | *553.30* | *4.21* |
| *LightCNN [16]* | *87.57* | *87.55* | *87.57* | *87.52* | *461.11* | *1.32* |
| *MobileNet* | *84.81* | *85.60* | *84.81* | *84.50* | *350.27* | *1.00* |
| *MobileNetV2* | *85.08* | *85.59* | *85.08* | *84.88* | *385.75* | *1.05* |
| *NAS_m1 [30]* | *86.46* | *86.47* | *86.46* | *86.45* | *399.68* | *1.30* |
| *NAS_m2 [30]* | *86.46* | *86.53* | *86.46* | *86.44* | *410.72* | *1.31* |
| *NASNetMobile* | *86.74* | *86.70* | *86.74* | *86.68* | *406.42* | *1.34* |
| *Proposed (LiteFeatNet)* | ***90.33*** | ***90.69*** | ***90.33*** | ***90.27*** | ***409.35*** | ***1.32*** |
| *Swin V2-Small* | *82.04* | *85.50* | *82.04* | *81.67* | *498.06* | *3.35* |
| *Swin V2-Tiny* | *82.04* | *83.74* | *82.04* | *81.83* | *252.16* | *2.15* |

**4.5 Class-wise performance metrics**

**Line # 788**

***Table 6. Class-wise performance metrics for RFMiD.***

| ***Model*** | ***Class*** | ***Accuracy*** | ***Precision*** | ***Sensitivity*** | ***Specificity*** |
| --- | --- | --- | --- | --- | --- |
| *CNN-1 [17]* | *DR* | *79.83* | *76.84* | *58.87* | *90.76* |
|  | *MH* | *84.25* | *75.27* | *67.31* | *91.08* |
|  | *NL* | *78.45* | *66.09* | *85.82* | *74.12* |
| *DenseNet121* | *DR* | *91.71* | *95.19* | *79.84* | *97.90* |
|  | *MH* | *93.37* | *87.04* | *90.38* | *94.57* |
|  | *NL* | *93.37* | *86.67* | *97.01* | *91.23* |
| *DeB5-XNet [24]* | *DR* | *91.99* | *93.58* | *82.26* | *97.06* |
|  | *MH* | *93.37* | *90.82* | *85.58* | *96.51* |
|  | *NL* | *91.99* | *83.87* | *97.01* | *89.03* |
| *DIA-VXNET [29]* | *DR* | *91.71* | *93.52* | *81.45* | *97.06* |
|  | *MH* | *92.54* | *88.12* | *85.58* | *95.35* |
|  | *NL* | *92.54* | *84.97* | *97.01* | *89.91* |
| *LightCNN [16]* | *DR* | *90.05* | *87.29* | *83.06* | *93.70* |
|  | *MH* | *92.26* | *86.54* | *86.54* | *94.57* |
|  | *NL* | *92.82* | *88.57* | *92.54* | *92.98* |
| *MobileNet* | *DR* | *87.57* | *90.72* | *70.97* | *96.22* |
|  | *MH* | *91.99* | *86.41* | *85.58* | *94.57* |
|  | *NL* | *90.05* | *80.25* | *97.01* | *85.96* |
| *MobileNetV2* | *DR* | *87.84* | *83.90* | *79.84* | *92.02* |
|  | *MH* | *91.16* | *91.86* | *75.96* | *97.29* |
|  | *NL* | *91.16* | *82.28* | *97.01* | *87.72* |
| *NAS_m1 [30]* | *DR* | *88.95* | *83.33* | *84.68* | *91.18* |
|  | *MH* | *91.71* | *87.00* | *83.65* | *94.96* |
|  | *NL* | *92.26* | *88.97* | *90.30* | *93.42* |
| *NAS_m2 [30]* | *DR* | *89.50* | *83.59* | *86.29* | *91.18* |
|  | *MH* | *91.71* | *88.54* | *81.73* | *95.74* |
|  | *NL* | *91.71* | *87.68* | *90.30* | *92.54* |
| *NASNetMobile* | *DR* | *89.23* | *85.71* | *82.26* | *92.86* |
|  | *MH* | *91.71* | *86.27* | *84.61* | *94.57* |
|  | *NL* | *92.54* | *87.94* | *92.54* | *92.54* |
| *Proposed (LiteFeatNet)* | *DR* | ***92.54*** | ***95.33*** | ***82.26*** | ***97.90*** |
|  | *MH* | ***92.54*** | ***84.07*** | ***91.35*** | ***93.02*** |
|  | *NL* | ***95.58*** | ***91.55*** | ***97.01*** | ***94.74*** |
| *Swin V2-Small* | *DR* | *87.29* | *98.75* | *63.71* | *99.58* |
|  | *MH* | *91.16* | *86.73* | *81.73* | *94.96* |
|  | *NL* | *85.63* | *72.28* | *99.25* | *77.63* |
| *Swin V2-Tiny* | *DR* | *86.19* | *83.64* | *74.19* | *92.44* |
|  | *MH* | *90.88* | *94.94* | *72.11* | *98.45* |
|  | *NL* | *87.02* | *75.14* | *97.01* | *81.14* |

**5.5 Evaluation metrics**

**Line # 925**

***Table 7. Evaluation metrics for RFMiD 2.0.***

| ***Model*** | ***Accuracy*** | ***Precision*** | ***Recall*** | ***F1-Score*** | ***Inference Time*** |
| --- | --- | --- | --- | --- | --- |
| *CNN-1 [17]* | *87.10* | *91.45* | *87.10* | *88.38* | *0.93* |
| *DenseNet121* | *94.09* | *94.58* | *94.09* | *94.24* | *1.60* |
| *DeB5-XNet [24]* | *93.28* | *93.89* | *93.28* | *93.47* | *2.90* |
| *DIA-VXNET [29]* | *92.74* | *94.31* | *92.74* | *93.12* | *4.04* |
| *LightCNN [16]* | *93.82* | *94.04* | *93.82* | *93.90* | *1.36* |
| *MobileNet* | *92.47* | *92.77* | *92.47* | *92.47* | *1.04* |
| *MobileNetV2* | *91.93* | *92.07* | *91.93* | *91.95* | *1.07* |
| *NAS_m1 [30]* | *92.47* | *92.92* | *92.47* | *92.57* | *1.33* |
| *NAS_m2 [30]* | *90.32* | *90.88* | *90.32* | *90.48* | *1.33* |
| *NASNetMobile* | *93.28* | *93.48* | *93.28* | *93.35* | *1.36* |
| *Proposed (LiteFeatNet)* | ***93.55*** | ***94.12*** | ***93.55*** | ***93.73*** | ***1.33*** |
| *Swin V2-Small* | *91.67* | *91.13* | *91.67* | *91.22* | *3.32* |
| *Swin V2-Tiny* | *94.89* | *94.68* | *94.89* | *94.74* | *2.02* |

**# 4**

The authors utilize only 3 of the 46 available disease categories in the RFMiD dataset (DR, MH, and Normal). Several questions arise:

- What criteria drove the selection of these specific categories?

- Can we reasonably expect the DIFE and PFMR approach to generalize to the remaining 43 disease categories, or to a larger multi-class problem?

The limited scope constrains the claims of clinical utility and generalizability.

**Response**

**What criteria drove the selection of these specific categories?**

We sincerely thank the reviewer for careful reading of our manuscript and for raising this insightful question regarding the selection of categories. We also appreciate the reviewer for providing us with the opportunity to clarify our rationale. We confirm that the choice of disease categories was not arbitrary. Instead, our choice was fact-driven, logical, and scientifically valid. These facts are stated below:

RFMiD exhibits substantial class imbalance with several class labels having only a very few samples (below 50 and even below 10). The selected categories for our study: Diabetic Retinopathy (DR), Media Haze (MH), and Normal (NL) are the well-represented classes with having sufficient number of images across the training, validation, and testing splits. The diseases such as DR and MH also have high clinical relevance and practical significance. DR is progressive and leading cause of vision loss worldwide affecting a high number of patients worldwide. Developing countries often face challenges such as limited availability of experienced ophthalmologists and lower diagnosis time per patient for managing the ever-increasing patient count, sometimes leading to misdiagnosis. This sets stage for accurate and reliable automated disease identification. MH is also common in retinal imaging and results in media opacities, thus poses a challenge for automated disease identification from fundus images. So, we selected it to ensure the robustness of our model to variations in image quality. NL images were included for enabling model to distinguish between healthy retinal structures from diseased pathologies. Therefore, we selected these categories due to the following criteria to assess the effectiveness of our proposed methodology.

1. To have clinical and practical significance.
2. To achieve stable model training backed with adequate training samples.
3. To enable reliable performance evaluation.
4. To ensure model’s effectiveness in handling images with varying quality.

We believe that our clarification regarding the choice of category selection strengthens clinical relevance, methodological rigor, transparency, and overall robustness of our study. To further clarify this, we have revised our manuscript in various sections, as follows:

**1 Introduction**

**Line # 56-60**

*Among various retinal diseases, Diabetic Retinopathy (DR), being common and progressive, is one of the leading causes of mild to severe vision impairments that may lead to irreversible damage as well as blindness if left untreated. Media Haze (MH) causes opacities in the eye’s optical media lead to blurry vision, and it is also quite common worldwide.*

**3.1 Dataset description**

**Line # 335-356**

*RFMiD is inherently an imbalanced dataset, with several classes having very few samples (typically below 50 or even 10). It also contains well-represented categories such as DR, MH, and NL, with much higher sample counts (632 for DR, 523 for MH, and 669 for NL). These retinal pathologies were selected for this study, and the selection criteria for these categories were fact-driven and guided by numerous clinical, practical, statistical, and experimental factors.*

*From both clinical and practical perspectives, DR and MH are relatively common diseases affecting large populations worldwide. As discussed earlier [1], developing countries face numerous challenges due to limited resources, which may sometimes lead to misdiagnosis. These challenges require reliable automated Computer-Aided Diagnosis (CAD) systems for effective and efficient patient management through accurate and timely diagnosis. MH, on the other hand, causes media opacities such as cataract, making it difficult to capture high-quality images of retinal structures. Images from the NL class label were selected to evaluate the effectiveness of our proposed model in handling fundus images with varying quality. This also ensures the model’s ability to distinguish healthy retinas from diseased ones, thereby reducing false diagnoses of healthy patients. Sufficient sample counts for these categories is an important statistical factor that ensures stability in training and reliability in evaluating the proposed model. The selection of class labels, thus performed, also enabled us to simulate a controlled experimental setting for rigorously evaluating our proposed architecture.*

**Can we reasonably expect the DIFE and PFMR approach to generalize to the remaining 43 disease categories, or to a larger multi-class problem?**

We are grateful to the reviewer for this important observation regarding the generalization and scalability of our proposed framework, particularly for a larger multi-class problem. Therefore, to evaluate this, we conducted additional experiments using two more common retinal pathologies: Macular Scar (MS) and Myopia (MYA) from the original RFMiD. To overcome severe class imbalance, we supplemented images from another publicly available multi-class retinal dataset. Experimental evaluation was performed using two different scenarios:

**Scenario # 1:** 4-class labels

**Scenario # 2:** 5-class labels

Our proposed framework demonstrated a strong and stable performance (accuracy: 88.86% and 85.42% for 4- and 5-class scenarios, respectively) for both experimental scenarios. To provide detailed insights, we also compared its performance with several baseline architectures. These baseline models showed notable performance degradation (particularly from 4-class scenario to 5-class scenario) whereas our proposed model only exhibited a modest performance drop. Importantly, our proposed model achieved competitive performance and maintained significantly lower inference time.

These results provide empirical evidence that our proposed framework is not limited to only a small subset of disease (i.e. DR, MH, and NL from RFMiD). It is robust enough in effectively generalizing to 4-class and 5-class scenarios as well. Therefore, we can reasonably expect the DIFE and PFMR approach to generalize to larger multi-class classification problems.

We have added a new section **(6. Scalability assessment)** in our manuscript containing all the necessary experimental protocol followed, results, and discussion. Amendments are also made to various other sections of the manuscript due to this valuable addition in our manuscript. The updates, thus made, are as follows:

**Abstract**

**Line # 35-39**

*To evaluate the scalability and adaptability of our proposed framework for larger multi-class problems, we assessed the scalability by increasing class label complexity using two additional disease categories. Results validated the effectiveness and computational efficiency of the proposed architecture compared with 9 baseline architectures.*

**1 Introduction**

**Line # 152-155**

*Sections 5, 6, and 7 present well-structured generalizability, scalability assessment, and ablation studies of the proposed model, whereas Section 8 showcases the conclusion, limitations, and future work of this study.*

**1.1 Research contribution**

**Line # 140-141**

*5) Evaluate the scalability, adaptability, and robustness of architectural components for larger multi-class problems.*

**6 Scalability assessment**

**Line # 985-1079**

*To further evaluate the scalability of the proposed framework, we extended our original 3-class classification problem. We conducted additional experiments using increasingly complex multi-class classification scenarios with 4 and 5 class labels, respectively. To perform this extension, we added two new classes to the original 3-class dataset to introduce additional inter-class complexity, which is critical for assessing the robustness of deep learning models. For each additional disease class, we have selected a common and underrepresented retinal pathology from the RFMiD. These retinal disease classes included Macular Scar (MS) and Myopia (MYA) having 27 and 167 images in RFMiD, respectively. To overcome the class imbalance, given our 3-class problem, we supplemented these additional categories with images from a third publicly available external dataset (accessed on 20 February 2026), collected from Anawara Hamida Eye Hospital and B. N. S. B. Zahurul Haque Eye Hospital. This dataset contains 5,335 images corresponding to 9 retinal pathologies [63–65]. Fig 14 shows the distribution of dataset samples across various retinal pathologies.*

*Fig 14. External Dataset for Scalability Assessment.*

*To upsample the underrepresented classes MS and MYA in the RFMiD, samples for these categories were extracted from this third dataset. To merge these samples with RFMiD, we split the extracted data samples into a train-validate-test ratio of 60:20:20 independently using stratified random sampling. In a recent study, [66] utilized image samples of two classes from a label-rich dataset.*

*The extracted samples were merged with the dataset used in our original 3-class problem. Special care was taken to prevent data leakage problem during merging, as both these datasets were collected from different geographical locations. Table 8 presents the contribution of images from both datasets for this scalability study.*

*Table 8. Class labels and the number of images supplemented for scalability assessment.*

| *Multi-class problem*  I.  | *RFMiD* | | *Anawara Hamida and B. N. S. B. Zahurul Haque Eye Hospitals [63–65]* | | *Total Number of supplemented images* |
| --- | --- | --- | --- | --- | --- |
|  | *MS* | *MYA* | *MS* | *MYA* |  |
| *4-class* | *27* | *-* | *444* | *-* | *471* |
| *5-class* | *27* | *167* | *444* | *500* | *1138* |

*We conducted numerous experiments and compared our proposed architecture with 9 state-of-the-art baseline models: Low-Cost CNN [7], CNN-2 [15], LightCNN [16], CNN-1 [17], CNN-3 [18], DeB5-XNet [24], DIA-VXNET [29], NAS_m1 [30], and NAS_m2 [30] to evaluate the scalability. It is worth mentioning that we used a consistent dataset pre-processing, augmentation pipeline, and training/evaluation setup, as in our original three class experiments.*

*To evaluate the performance of numerous architectures, we have considered accuracy, precision, recall, F1-score, and inference time as evaluation metrics. The overall performance metrics for the 4-class and 5-class problems are shown in Table 9 and Table 10, respectively.*

*Table 9. Performance metrics for 4 retinal pathologies.*

| ***Model*** | ***Accuracy*** | ***Precision*** | ***Recall*** | ***F1-Score*** | ***Inference Time*** |
| --- | --- | --- | --- | --- | --- |
| *CNN-1 [17]* | *78.82* | *79.66* | *78.82* | *78.67* | *1.03* |
| *CNN-2 [15]* | *74.02* | *75.63* | *74.02* | *73.85* | *1.05* |
| *CNN-3 [18]* | *82.10* | *82.60* | *82.10* | *81.99* | *0.93* |
| *DeB5-XNet [24]* | *89.74* | *90.17* | *89.74* | *89.72* | *3.73* |
| *DIA-VXNET [29]* | *87.34* | *88.03* | *87.34* | *87.26* | *5.71* |
| *LightCNN [16]* | *87.77* | *87.95* | *87.77* | *87.73* | *1.54* |
| *Low-Cost CNN [7]* | *79.04* | *79.91* | *79.04* | *79.06* | *4.40* |
| *NAS_m1 [30]* | *84.28* | *84.46* | *84.28* | *84.27* | *1.58* |
| *NAS_m2 [30]* | *83.84* | *83.40* | *83.84* | *83.79* | *1.61* |
| *Proposed (LiteFeatNet)* | ***88.86*** | ***89.06*** | ***88.86*** | ***88.85*** | ***1.55*** |

*Accuracy, Precision, Recall, and F1-Score (%), and Inference Time (seconds)*

*Table 10. Performance metrics for 5 retinal pathologies.*

| ***Model*** | ***Accuracy*** | ***Precision*** | ***Recall*** | ***F1-Score*** | ***Inference Time*** |
| --- | --- | --- | --- | --- | --- |
| *CNN-1 [17]* | *70.51* | *72.00* | *70.51* | *70.32* | *1.31* |
| *CNN-2 [15]* | *65.59* | *68.07* | *65.59* | *65.14* | *1.32* |
| *CNN-3 [18]* | *73.39* | *76.78* | *73.39* | *73.29* | *1.21* |
| *DeB5-XNet [24]* | *87.46* | *87.97* | *87.46* | *87.33* | *4.44* |
| *DIA-VXNET [29]* | *87.63* | *87.85* | *87.63* | *87.56* | *8.28* |
| *LightCNN [16]* | *85.08* | *85.87* | *85.08* | *85.18* | *1.97* |
| *Low-Cost CNN [7]* | *74.24* | *74.94* | *74.24* | *74.22* | *5.71* |
| *NAS_m1 [30]* | *80.85* | *81.41* | *80.85* | *80.97* | *1.93* |
| *NAS_m2 [30]* | *84.74* | *85.10* | *84.74* | *84.79* | *1.93* |
| *Proposed (LiteFeatNet)* | *85.42* | *86.52* | *85.42* | *85.53* | *1.95* |

*Accuracy, Precision, Recall, and F1-Score (%), and Inference Time (seconds)*

*As presented in Table 9, the proposed model demonstrated an accuracy (88.86%), precision (89.06%), recall (88.86%), and F1-score (88.85%), thereby making its place among the top two architectures. Among various compared architectures, DeB5-XNet [24] demonstrated the highest performance (accuracy: 89.74%, precision: 90.17%, recall: recall: 89.74%, and F1-score: 89.72%), but at a significantly higher inference time of 3.73 seconds compared with the proposed framework. Similarly, the architectures such as LightCNN [16] and DIA-VXNET [29] also achieved competitive accuracy scores of 87.77% and 87.34%, respectively with DIA-VXNET [29] having the highest inference time of 5.71 seconds among all baseline architectures. In contrast, the LiteFeatNet exhibited the second-best performance metrics, with a considerably lower testing time of 1.55 seconds compared with DeB5-XNet [24], LightCNN [16], and DIA-VXNET [29]. Our proposed model balances the classification performance and computational efficiency compared with NASNetMobile-based lightweight architectures NAS_m1 [30] and NAS_m2 [30]. On the other hand, the CNN architectures such as CNN-1 [17], CNN-2 [15], CNN-3 [18], and Low-Cost CNN [7], despite having much lower inference time, achieved considerably lower performance with accuracy scores ranging from 74% to 82%. This clearly indicates that these architectures struggled to learn pathological features for this classification problem with four distinct categories. In contrast, our proposed architecture captured disease-relevant features effectively and efficiently through the synergistic combination of DIFE, PFMR and LCC modules.*

*Table 10 summarizes the performance of various architectures for another larger multi-class scenario having 5 categories. According to Table 10, architectures such as CNN-1 [17], CNN-2 [15], and CNN-3 [18] experienced substantial performance drops from 4-class scenario, with accuracy scores of 70.51%, 65.59%, and 73.39%, respectively. This performance once again highlights the limitation of these architectures in capturing complex pathological features. On the other hand, the architectures NAS_m2 [30], NAS_m1 [30], and Low-Cost CNN [7] improved classification performance, achieving accuracies of 84.74%, 80.85%, and 74.24%, respectively. Despite the lower inference time (1.93 seconds), both NAS_m2 [30] and NAS_m1 [30] underperformed the top 3 best-performing architectures: DIA-VXNET [29], DeB5-XNet [24], and the proposed LiteFeatNet architecture.*

*The architectures DIA-VXNET [29] and DeB5-XNet [24], being top-ranked, demonstrated strong discriminative capabilities with performance (accuracy: 87.63% vs. 87.46%, precision: 87.85% vs. 87.97%, recall: 87.63% vs. 87.46%, and F1-score: 87.56% vs. 87.33%). However, both these architectures were computationally costly with higher inference times (8.28 vs. 4.44 seconds). This increased testing time may limit their real-time clinical implementation, particularly in scenarios with higher patient count, which are common worldwide. In comparison, our proposed architecture successfully maintained competitive predictive power, achieving an accuracy of 85.42%, precision of 86.52%, recall of 85.42%, and F1-score of 85.53%, while demonstrating a low computational overhead with 1.95 seconds as inference time. Moreover, the performance degradation of our proposed model from a 4-class to a 5-class scenario was minimal, thereby validating the robustness and scalability of the proposed LiteFeatNet, even with increasing class label complexity.*

*Overall, the proposed architecture demonstrated robust predictive performance and outperformed numerous state-of-the-art baseline models, thereby indicating its superior feature extraction and learning. Moreover, its minimal performance reduction, when scaling from a 4-class to a 5-class scenario, highlights LiteFeatNet’s generalizability to more diverse label spaces. The results also validate that the integration of the architectural components (DIFE, PFMR, and LCC) has enabled our proposed framework to be robust, scalable, adaptable, and computationally efficient, beyond the original 3-class experimental scenario, thereby positioning it as a viable automated diagnostic tool suitable for larger multi-class problems in real-world clinical deployment.*

**# 5**

The authors place considerable emphasis on training time as a metric of efficiency (e.g., "6.82 minutes of training"). However, training computational cost has minimal bearing on deployment feasibility in resource-constrained environments. Models are trained once, typically on capable hardware, making it acceptable, even advisable, to invest substantial time at this stage. The relevant metrics for deployment are model size and inference-time computational cost. The manuscript would benefit from refocusing this discussion accordingly.

**Response**

We cordially thank the reviewer for this insightful observation. We fully agree with training time’s limited relevancy with the real-time deployment and operations, as models are typically trained using capable hardware resources. Therefore, we have updated the manuscript and placed more emphasis on the metrics such as model parameters, size and inference time, which are relevant ones for deployment. Various sections / sub-sections have been updated for refocusing our discussion towards this important point. The amendments, thus made, are as follows:

**Abstract**

**Line # 30-32**

*It outperformed twelve state-of-the-art models, achieving the highest testing accuracy of 90.33%, precision of 90.69%, recall of 90.33%, and F1-score of 90.27%, with a fast, impressive inference time of 4 milliseconds per image.*

**1 Introduction**

**Line # 108-110**

*In this study, a performance-centric, computationally efficient, and robust deep learning model is proposed that leverages transfer learning for accurate diagnosis of multiple retinal conditions.*

**Line # 114-116**

*The proposed architecture achieved the highest accuracy with fewer trainable parameters, a smaller model size, and computationally efficient deployment.*

**Line # 120-126**

*Generalization (on an external dataset), scalability assessment, and ablation studies also validate its suitability for deployment in resource-constrained clinical environments such as portable retinal scanning systems and mid-range smartphones. Such environments typically have limited memory, making it difficult for AI-assisted inference to run smoothly. Usage of compact models such as our proposed architecture makes model storage and loading easier, enabling reliable, memory-efficient, and fast inference.*

**4 Results and discussion**

**Line # 520-522**

*Moreover, to reflect development- and deployment-level computational efficiencies, we have included training and testing/inference times, in addition to the above-mentioned performance metrics.*

**4.4 Overall performance metrics**

**Line # 731-753**

*According to Table 5, the computational times of NASNetMobile (inference time: 1.34 seconds and training time: 406.42 seconds), NAS_m1 (inference time: 1.30 seconds and training time: 399.68 seconds) [30], NAS_m2 (inference time: 1.31 seconds and training time: 410.72 seconds) [30], and LiteFeatNet (inference time: 1.32 seconds and training time: 409.35 seconds) were quite close. The proposed architecture outperformed these models, with an impressive inference time, which is modestly higher, particularly compared to NAS_m1 [30] and NAS_m2 [30]. MobileNet had the lowest testing time (1.00 seconds), while Swin V2-Small had the longest inference and training times (3.35 and 498.06 seconds, respectively) among all the pre-trained architectures studied.*

*Both ensemble architectures, DeB5-XNet [24] and DIA-VXNET [29], required extended inference time (2.96 and 4.21 seconds, respectively), with DeB5-XNet [24] and DIA-VXNET [29] exhibiting the slowest training time (635.35 seconds) and inference time (4.21 seconds), respectively, among the customized architectures.*

*Although the inference and training times for the proposed model were considerably higher than those of CNN-1 [17] (1.32 vs. 0.91 seconds) and (409.35 vs. 325.65 seconds), the LiteFeatNet model achieved far better performance metrics with competitive computational efficiency. Moreover, LiteFeatNet, despite having fewer parameters, is substantially more efficient than both DeB5-XNet [24] and DIA-VXNET [29], offering much faster inference speed (1.32 vs. 2.96 seconds) when compared with DeB5-XNet [24]. The performance metrics suggest that LiteFeatNet is not only lightweight but also efficient and more reliable for retinal disease classification.*

**Line # 758-772**

*In addition to this performance gain (accuracy: 1.23%, precision: 1%, recall: 1.23%, and F1-score: 1.30%), the proposed LiteFeatNet also exhibits deployment and development computational efficiencies, with a lower inference time (1.32 vs. 1.59 seconds) and fast-paced training (409.35 vs. 416.62 seconds). Furthermore, the proposed framework also offers 30% (approximately) reduced memory footprint compared to DenseNet121. This modest size reduction is extremely important for deploying in real-world clinical settings. Apart from compact storage requirements, small models require less time to load and generate faster inferences, making them more suitable for integration with resource-scarce clinical settings, mass retinal scanning programs, and other situations requiring swift operations. Such practical scenarios rely on portable diagnostic tools, low-resourced CAD systems, and edge devices. In such real-time scenarios, models having fewer parameters, limited data movements, and cache optimization are usually preferred choice for integration because of their minimal impact on the device responsiveness [59].*

**Line 777-783**

*The results verify that the LiteFeatNet possesses superior capability to learn disease-wise discriminative features. Moreover, incorporating DIFE from the backbone, an optimized feature refinement strategy (PFMR), and an LCC module enabled the model to achieve robust performance for classifying NL, DR, and MH, with reduced computational cost, particularly the inference time (1.32 seconds), making it a suitable choice for deployment in resource-scarce environments, particularly with a higher patient counts.*

**5.5 Evaluation metrics**

**Line # 932-941**

*Among the pre-trained CNN models, DenseNet121 reported the highest accuracy (94.09%), precision (94.58%), recall (94.09%), and F1-score (94.24%). Although DenseNet121 outperformed the proposed model, its inference time (1.60 seconds) is significantly higher than that of LiteFeatNet (1.33 seconds). Thus, despite a minor performance reduction (accuracy: 0.57%, precision: 0.49%, recall: 0.57% and F1-score: 0.54%) compared with DenseNet121, our model still demonstrates deployment level efficiency (memory and time) on this external dataset. Lightweight architectures like MobileNet achieved competitive performance with a test time of 1.04 seconds, yet LiteFeatNet architecture outperformed it by achieving higher values of performance metrics.*

Line # 946-950

*However, both Swin V2-Small and Swin V2-Tiny have much longer inference times (3.32 and 2.02 seconds), which are significantly higher than the proposed architecture (1.33 seconds), thereby validating its superior deployment-level efficiency and predictive power.*

Line # 953-956

*Interestingly, LiteFeatNet achieved better precision (94.12% vs. 94.04%) while requiring less inference time as compared to LightCNN's [16] (1.36 seconds), suggesting a well-balanced predictive power, efficient parameter-utilization, and faster operational time.*

Line # 963-965

*However, both ensemble architectures take much longer (2.90 and 4.04 seconds) to predict class labels, validating LiteFeatNet's efficiency, effectiveness, and robustness, specifically on resource-constrained environments.*

Line # 978-983

*Its performance on RFMiD 2.0 was excellent compared with numerous state-of-the-art architectures, considering its parameter size and computational cost (inference time: 0.004 seconds/image), making it a strong prospect for practical deployment in the healthcare sector requiring efficient usage of the available resources, portable devices, and low-resourced CAD systems for effectively managing the increasing patient count.*

6 Scalability evaluation

Line # 1034-1038

*In contrast, the LiteFeatNet exhibited the second-best performance metrics, with a considerably lower testing time of 1.55 seconds compared with DeB5-XNet [24], LightCNN [16], and DIA-VXNET [29]. Our proposed model balances the classification performance and computational efficiency compared with NASNetMobile-based lightweight architectures NAS_m1 [30] and NAS_m2 [30].*

Line # 1063-1067

*In comparison, our proposed architecture successfully maintained competitive predictive power, achieving an accuracy of 85.42%, precision of 86.52%, recall of 85.42%, and F1-score of 85.53%, while demonstrating a low computational overhead with 1.95 seconds as inference time.*

Line # 1074-1079

*The results also validate that the integration of the architectural components (DIFE, PFMR, and LCC) has enabled our proposed framework to be robust, scalable, adaptable, and computationally efficient, beyond the original 3-class experimental scenario, thereby positioning it as a viable automated diagnostic tool suitable for larger multi-class problems in real-world clinical deployment.*

7.1 Ablation experiments for the proposed framework

Line # 1122-1126

*Nevertheless, the LiteFeatNetA derives its substantial benefits and predictive strength from the synergistic combination of DIFE and PFMR, confirming their complementary strengths and roles in designing a memory- and time-efficient, reliable deep neural network architecture for classifying multiple retinal pathologies effectively.*

Line # 1132-1134

*This configuration takes 1.27 seconds to generate an inference for 362 test images, which is lowest among other ablation experiments with the proposed framework.*

Line # 1144-1145

*The LiteFeatNetA model slightly increased the testing time to 1.32 seconds, which is fully justified by the integration of DIFE, PFMR and LCC modules.*

Line # 1152-1154

*Hence, this ablation study strongly validates that both DIFE and PFMR contribute implicitly to achieving the optimal classification performance while maintaining computational efficiency.*

Conclusions

Line # 1247-1249

*Meanwhile, the LiteFeatNet model significantly reduced the number of parameters, associated model size, and computational efficiency, particularly the inference time.*

Line # 1254-1259

*The findings from experiments suggest that LiteFeatNet, powered by memory-optimized and time-efficient low-learning, has proven that it can maintain higher accuracy and sensitivity without sacrificing sensitivity, making it a suitable choice for deployment in real-time, resource-constrained environments, particularly in underdeveloped regions, where both diagnostic reliability and computational resource efficiency are incredibly crucial.*

**# 6**

The authors describe a 60:40 train-evaluation split but report that evaluation was performed on a test set of 362 images. The manuscript should clarify the exact composition of the data splits. Specifically, the authors should confirm that:

(a) the validation set comprises only the 368 images mentioned,

(b) the test set of 362 images was held out and not used during model selection or hyperparameter tuning, and (c) there is no data leakage between splits.

**Response**

We appreciate reviewer for observing and pointing out this important point. We also truly believe that the there is a stronger need to clarify the exact composition of the dataset splits to improve quality and understanding of the manuscript. To clarify and explain this, we have explicitly included the term *“train-validate-test ratio”* instead of “train-test ratio” or “train-evaluate ratio” in numerous sections of this revised manuscript. Additionally, for absolute clarity, we have also mentioned *“60:20:20”* in place of “60:40” as *“train-validate-test ratio”* in this revised manuscript at two distinct locations **Abstract** and **Section 3.1 Dataset description**. We have used the following composition of data splits from RFMiD:

| **Dataset Split** | **No. of Images** |
| --- | --- |
| Training | 1,094 |
| Validation | 368 |
| Testing | 362 |
| ***Total*** | ***1,824*** |

The stats of the dataset composition are also included in the manuscript as **Fig 3**. For clarity, we have also amended the caption from “Class label distribution in the RFMiD.” to *“Class label distribution for the study.”*

We are also grateful to the reviewer for highlighting the need for clarification of various aspects related to dataset splits, images in the splits, and data leakage. We fully agree that the including the confirmation of validation split, usage of test split, and data leakage in the manuscript will truly improve the clarity of the methodology used in this study. Below are the details of brief explanation and amendments made in the manuscript in response to reviewer’s comments:

**Abstract**

**Line # 26-28**

*Experiments are conducted using 1824 images from three distinct class labels in the Retinal Fundus Multi-Disease Image Dataset (RFMiD), with a 60:20:20 train-validation-test split.*

**3.1 Dataset description**

**Line # 361-363**

*Out of 1824 images, the total numbers in the validation and test sets are 368 and 362, respectively, resulting in a 60:20:20 train-validation-test split.*

**Line # 363-365**

*Fig 3 presents the distribution of image samples and class labels used in this study.*

***Fig 3. Class label distribution for the study.***

(a) **the validation set comprises only the 368 images mentioned**

We truly acknowledge the need for the conformation and clarity regarding the images in the validation set. The validation set was utilized in this study comprises of only 368 images belonging to the chosen class labels. To further clarify this, we have explicitly included the details at multiple locations in this revised manuscript text. We have also explicitly included class-label-wise distribution of image samples across the training, validation, and testing splits in **Fig 3**.

**3.1 Dataset description**

**Line # 361-363**

*Out of 1824 images, the total numbers in the validation and test sets are 368 and 362, respectively, resulting in a 60:20:20 train-validation-test split.*

**Line # 363-365**

*Fig 3 presents the distribution of image samples and class labels used in this study.*

***Fig 3. Class label distribution for the study.***

**4 Results and discussion**

**Line # 514-517**

*The performance of the proposed model (LiteFeatNet) is evaluated using widely accepted confusion-matrix-driven classification metrics, including accuracy, precision, recall, specificity, and F1-score [51] on 362 images from the test set of the RFMiD.*

**7.1 Ablation experiments with the proposed framework**

**Line # 1132-1134**

*This configuration takes 1.27 seconds to generate an inference for 362 test images, which is lowest among other ablation experiments with the proposed framework.*

**(b) the test set of 362 images was held out and not used during model selection or hyperparameter tuning**

We appreciate the reviewer for this valuable suggestion regarding the test set images. We utilized three independent dataset splits (training, validation, and test) in this study. The test set (with 362 images) was strictly held out during the model development. It was not used for model selection, hyper parameter tuning, or any training-related procedure. We only used the test set for testing/final evaluation of the performance. To provide factual details to readers we have made a number of amendments is this revised manuscript, as follows:

**3.1 Dataset description**

**Line # 357-363**

*The RFMiD dataset is divided into pre-defined training, validation, and testing splits by the original contributors.* *To evaluate our proposed framework, 1824 images for these class labels are selected directly from the predefined sub-sets, without structural amendments to the original dataset, re-splitting, or biased sample selections. This experimental protocol ensured that there was no data leakage across these splits. Out of 1824 images, the total numbers in the validation and test sets are 368 and 362, respectively, resulting in a 60:20:20 train-validation-test split.*

**Line # 366-368**

*It is worth mentioning that both validation and test sets were non-overlapping and independent from each other. All the models used in this study were trained using 1094 training set samples, which are independent from validation and test sets.*

(c) **there is no data leakage between splits**

We are grateful to the reviewer for raising this important concern regarding the dataset composition and experimental protocol. We apologize for the lack of clarity in the submitted manuscript and we also believe that providing details on data leakage concern exclusively will truly improve the clarity in our methodology.

The dataset used in this study was already divided into three splits: training (60%, 1920 images), validation (20%, 640 images), and testing (20%, 640 images) splits. According to the dataset descriptor, the original contributors have clearly mentioned that disease-wise stratification was maintained across these splits. In our study, we strictly adhered to these partitions and did not perform any structural changes or re-splitting to the dataset. All the images used in this study were extracted directly from the pre-defined splits while preserving the original preservation. Therefore, we confirm that there was no data leakage between these splits, as we ensured that original splitting, as defined by original contributors, was followed for experiments.

To solve this ambiguity we have revised our manuscript, as follows:

**3.1 Dataset description**

**Line # 357-363**

*The RFMiD dataset is divided into pre-defined training, validation, and testing splits by the original contributors.* *To evaluate our proposed framework, 1824 images for these class labels are selected directly from the predefined sub-sets, without structural amendments to the original dataset, re-splitting, or biased sample selections. This experimental protocol ensured that there was no data leakage across these splits. Out of 1824 images, the total numbers in the validation and test sets are 368 and 362, respectively, resulting in a 60:20:20 train-validation-test split.*

We believe that these clarifications resolve the concerns raised by the reviewer. Moreover, the amendments made in response improve the clarity, transparency, and reproducibility of this study.

**Minor Concerns:**

**# 1**

The ablation study convincingly demonstrates the synergistic benefit of DIFE and PFMR. However, the authors only evaluate these components with the NASNetMobile backbone. Given the promising results, did the authors consider implementing DIFE and PFMR with other backbone architectures (e.g., MobileNetV2, DenseNet121)? This would strengthen claims about the general applicability of the proposed approach and would be a natural extension given the ablation findings.

**Response**

We appreciate the reviewer for recognizing the quality of the ablation study and pointing out an important concern regarding integration of these modules with other backbone architectures. We also like to thank reviewer for providing us with valuable suggestions and opportunities that will surely strengthen claims of the general applicability of our proposed approach by extending the ablation findings. To do this, we have conducted a detailed ablation study by using 8 different ablation models, utilizing DenseNet121 and MobileNetV2 architectures. The results assure the effectiveness and synergistic benefit of DIFE and PFMR for these architectures as well. We have also added a new sub-section **(sub-section 7.2)** in this revised manuscript to explain experimental protocol followed, results, and related discussion.

**Abstract**

**Line # 39-43**

*An ablation study was also conducted using our proposed LiteFeatNet and two top-performing transfer learning architectures to validate that the synergistic combination of deep feature extraction and feature map refinement is the primary design decision behind the success of the LiteFeatNet architecture.*

**7 Ablation study**

**Line # 1082-1084**

*To obtain detailed insights into the integration of DIFE and PFMR, we conducted ablation experiments for our proposed framework and two backbone architectures.*

**7.2 Ablation experiments for other backbone architectures**

**Line # 1160-1236**

*We also evaluated the impact of DIFE and PFMR with two top-performing backbone feature extraction architectures: DenseNet121 and MobileNetV2. To conduct these ablation experiments, we evaluated eight model variants, four for each DenseNet121 and MobileNetV2 backbone feature extractor. The suffixes (A, B, C, and D) denote the consistency in ablation model configurations. The architectural specifications used in these ablation experiments and corresponding evaluation metrics for this second set of experiments are shown in Table 12.*

*Table 12. Ablation experiments for other backbone architectures.*

| *Ablation Model* | *DIFE* | *PFMR* | *Accuracy* | *Precision* | *Recall* | *F1-Score* | *Training Time* | *Inference Time* |
| --- | --- | --- | --- | --- | --- | --- | --- | --- |
| *DenseNet121A* | *✓* | *✓* | *89.78* | *90.21* | *89.78* | *89.71* | *401.93* | *1.57* |
| *DenseNet121B* | *✗* | *✗* | *88.40* | *89.25* | *88.40* | *88.33* | *420.59* | *1.46* |
| *DenseNet121C* | *✓* | *✗* | *89.23* | *90.00* | *89.23* | *89.15* | *401.31* | *1.43* |
| *DenseNet121D* | *✗* | *✓* | *89.50* | *89.87* | *89.50* | *89.44* | *413.68* | *1.53* |
| *MobileNetV2A* | *✓* | *✓* | *86.19* | *86.61* | *86.19* | *86.02* | *350.62* | *1.00* |
| *MobileNetV2B* | *✗* | *✗* | *83.98* | *84.58* | *83.98* | *83.72* | *343.02* | *0.98* |
| *MobileNetV2C* | *✓* | *✗* | *85.36* | *86.16* | *85.36* | *85.11* | *345.75* | *0.98* |
| *MobileNetV2D* | *✗* | *✓* | *86.19* | *86.57* | *86.19* | *86.01* | *361.12* | *1.01* |

*Accuracy, Precision, Recall, and F1-Score (%), Training Time, and Inference Time (seconds)*

*To provide a quantitative measure of the effectiveness of the DIFE, PFMR, and LCC modules, we have evaluated the improvement (%) in the performance of various ablation models by using their corresponding transfer learning architectures as baseline references. The percentage improvement/reduction in the performance of NASNetMobile, DenseNet121, and MobileNetV2, are presented in Fig 16, Fig 17, and Fig 18, respectively.*

***Fig 16. Performance improvement with NASNetMobile backbone.***

***Fig 17. Performance improvement with DenseNet121 backbone.***

***Fig 18. Performance improvement with MobileNetV2 backbone.***

*As shown in Fig 16, the LiteFeatNetB improved the accuracy, precision, recall, and F1-score by 0.96%, 0.91%, 0.96%, and 0.93%, respectively, compared with the reference fine-tuned NASNetMobile. Conversely, the same architectural configuration with DenseNet121 and MobileNetV2 backbones reported considerable performance reduction (accuracy: 0.93%, precision: 0.49%, recall: 0.93%, and F1-score: 0.88%) and (accuracy: 1.29%, precision: 1.18%, recall: 1.29%, and F1-score: 1.37%), as shown in Fig 17 and Fig 18, respectively, in comparison to the baseline transfer learning settings. These results effectively highlight the role of the LCC module in the performance improvement of the proposed framework. It is also revealed that integrating the LCC module solely with DenseNet121 and MobileNetV2 backbones results in performance drop, thereby limiting its effectiveness with these architectures, particularly when integrated alone.*

*As shown in Fig 16, the LiteFeatNetC model improved performance (accuracy: 1.59%, precision: 1.70%, recall: 1.59%, and F1-score: 1.57%) in comparison with the reference baseline. Whereas, according to Fig 17, DenseNet121C marginally increased baseline’s precision and F1-score of the by 0.35% and 0.04%, respectively. The MobileNetV2C, as shown in Fig 18, also reported a fractional performance improvement of 0.33% in accuracy, 0.67% in precision, 0.33% in recall, and 0.27% in F1-score, as compared with MobileNetV2. Thus, integration of the DIFE proved more beneficial for LiteFeatNet than the DenseNet121 and MobileNetV2 backbones, which again validates our design decision behind the proposed framework.*

*The model LiteFeatNetD improved the performance metrics of LiteFeatNetC by 2-fold (approximately), as shown in Fig 16. It achieved improvements of 3.18% in accuracy, 3.23% in precision, 3.18% in recall, and 3.20% in F1-score, compared with the NASNetMobile. As shown in Fig 17, DenseNet121D slightly improved all the performance metrics (accuracy: 0.30%, recall: 0.30%; precision: 0.20%; F1-score: 0.37%) compared to the baseline DenseNet121. Whereas, the configuration MobileNetV2D exhibited substantial improvement across all performance evaluation metrics (accuracy, recall, and F1-score: 1.30%; precision: 1.14%), as given in Fig 18, clearly validating the effectiveness of the PFMR module, particularly for lightweight architectures such as NASNetMobile and MobileNetV2.*

*Interestingly, all the DIFE-PFMR-integrated model variants, LiteFeatNetA, DenseNet121A, and MobileNetV2A, dramatically improved the performance of the corresponding baseline architectures. According to Fig 16, the performance of the NASNetMobile baseline was improved by a comprehensive margin (accuracy: 4.14%, precision: 4.60%, recall: 4.14%, and F1-score: 4.14%). Similarly, DenseNet121A also showed moderate improvements in accuracy (0.62%), precision (0.58%), recall (0.62%), and F1-score (0.67%), as shown in Fig 17. MobileNetV2A, though reported only a marginal increase in precision and F1-score compared with MobileNetV2D, demonstrated a substantial improvement of 1.30% in accuracy, 1.19% in precision, 1.30% in recall, and 1.34% in F1-score (as presented in Fig 18) compared with the baseline version. The performance metrics achieved confirm that the effectiveness of integrating DIFE and PFMR module across different backbones was superior when compared with other configurations.*

*Fig 19 compares the inference times of LiteFeatNetA, DenseNet121A, and MobileNetV2A with their counterparts. According to Fig 19, the ablation models MobileNetV2A, DenseNet121A, and LiteFeatNetA reported lower inference times (1.00, 1.57, and 1.32 seconds, respectively) than those of MobileNetV2, DenseNet121, and NASNetMobile (1.05, 1.59, and 1.34 seconds, respectively). This, once again confirms that the DIFE and PFMR synergy empowers transfer learning models to be computationally efficient, thereby enabling deployment-level efficiency in resource-constrained environments.*

***Fig 19. Comparison of inference times.***

*All results with the configuration utilizing both DIFE and PFMR confirm the synergistic effect of DIFE and PFMR for performance improvement across all the baselines, in particular. Moreover, their integration with NASNetMobile backbone demonstrated the effectiveness, efficiency, and motivation behind the specific architectural choices made for designing a lightweight, performance-centric, and computationally efficient LiteFeatNet architecture.*

**# 2**

The manuscript frequently references deployment in "resource-constrained environments" without quantitative specification. The authors should provide:

- Concrete examples of target deployment platforms (specific smartphone models, embedded systems, etc.)

- Memory and computational constraints of these platforms

- Threshold model sizes that these environments can reasonably host

This would help readers assess whether the 19.87 MB model size represents a meaningful improvement for practical deployment scenarios.

**Response**

We are really thankful to the reviewer for keenly observing and reviewing our manuscript, and raising this important concern. We also believe that updating our manuscript in the light of valuable suggestions by reviewer will represent a meaningful improvement for practical deployment scenarios. Therefore providing concrete examples of target platforms, constraints, and thresholds will truly improve the clarity and quality of our manuscript. In response to this concern, we have made a number of changes in this revised manuscript, which are as follows:

**4.1 Comparison of parameters and model size**

**Line # 608-627**

*The specifications of various resource-scarce target deployment platforms for the proposed model, along with resource consumption reported by numerous studies, are given in Table 4.*

***Table 4. Target deployment platforms.***

| ***Research study*** | ***Device / platform*** | ***Device specifications*** | ***Use case*** | ***Resource consumption reported*** |
| --- | --- | --- | --- | --- |
| *[53]* | *Raspberry Pi 4*  *Model B* | *Cortex A72 1.5 GHz (quad core), 4GB RAM (3200 MHz), No GPU* | *Image classification using CNN* | *512 MB of RAM* |
| *[53]* | *NVIDIA Jetson Nano* | *Cortex A57 1.43 GHz (quad core), 4GB RAM (1600 MHz), and Maxwell GPU with 128 cuda cores* | *Image classification using CNN* | *700 MB of RAM* |
| *[54]* | *Raspberry Pi 4* | *Cortex A72 1.5 GHz (quad core), 4GB RAM (3200 MHz), No GPU* | *Image classification using CNN* | *2.5 GB of RAM* |
| *[54]* | *NVIDIA Jetson Nano* | *Cortex A57 1.43 GHz (quad core), 4GB RAM (1600 MHz), and Maxwell GPU with 128 cuda cores* | *Image classification using CNN* | *3.5 GB of RAM* |
| *[55]* | *Xiaomi Black Shark* | *Qualcomm Snapdragon 845 octa-core 2.8GHz, 8GB RAM (1866 MHz), Qualcomm Adreno 630 GPU, 256 GB Storage* | *Object detection using YOLO v3* | *633 MB of RAM with OpenCV*  *263 MB of RAM with TensorFlow lite* |
| *[56]* | *Raspberry Pi 5*  *Model B Rev 1.0* | *Broadcom Cortex A76 2.4 GHz (quad-core), 8 GB RAM, VideoCore VII GPU, 64 GB Storage* | *Image classification using CNN* | *1948 MB of RAM* |
| *[56]* | *NVIDIA Jetson Nano 4.6.6* | *Cortex A57 (MP core), 4 GB RAM (1600 MHz), and Maxwell GPU with 128 cuda cores, 64 GB Storage* | *Image classification using CNN* | *1960 MB of RAM* |
| *[57]* | *Raspberry Pi 4*  *Model B* | *Cortex A72 1.5 GHz (quad core), 4GB RAM (3200 MHz), No GPU* | *Image classification using CNN* | *-* |

*The total memory M_t_ occupied by a model during inference is given by Eq (7), where M_p_ and M_a_ are the model’s parameters and activation memories, respectively, and M_o_ denotes the memory overhead. In the worst-case scenario (1024x1024 image size), M_a_ approximately reaches α = 16 times the M_p_ [58], defined by Eq (8).*

$M_{t}\approx M_{p}+M_{a}+M_{o}$	*(7)*

$M_{a}\approx{\alpha\times M}_{p}$	*(8)*

*Our proposed model (M_p_ = 19.87 MB) is estimated to require M_t_ = 337.79 + λ MB (M_o_ = λ) of RAM during inference with the worst-case scenario. However, for the standard 224x224 image size, M_a_ < M_p_ [58], and the inference memory for this size is always estimated to be below that for the worst-case scenario. Thus, the memory consumption threshold limits of the proposed model are given by Eq (9), where M_o_ = λ and M_a_ = β. Moreover, compared with the device specifications and memory consumptions reported in Table 4, our architecture is suitable for operating on the aforementioned resource-scarce computing devices, across image resolutions ranging from 224x224 to 1024x1024.*

$(19.87+\beta+ \lambda)\lesssim M_{t}\lesssim(337.79+ \lambda)$	*(9)*

**4.4 Overall performance metrics**

**Line # 774-776**

*Our proposed framework’s parameter count (5.2 million) and corresponding M_p_ effectively fall within this range, making M_t_ compatible with the deployment platforms given in Table 4.*

**# 3**

DenseNet121 achieves comparable evaluation performance to LiteFeatNet (89.23% vs. 90.33% accuracy), with the proposed model's primary advantage being approximately 30% smaller size (19.87 MB vs. 26.86 MB). The authors should more carefully contextualize this trade-off and discuss scenarios where this size reduction provides meaningful practical benefit.

**Response**

We are grateful to the reviewer for raising this thoughtful concern and valuable suggestion. We fully agree with the reviewer that contextualizing this tradeoff related to model size and performance will greatly benefit our manuscript. Moreover, discussing the practical benefits provided by this reduced size along with real life scenarios will improve the clarity and quality of our manuscript. Therefore, we have updated our manuscript for better contextualization of this trade-off, particularly in connection with the DenseNet121 architecture. The amendments made to this revised manuscript are as follows:

**4.4 Overall performance metrics**

**Line # 754-765**

*Interestingly, the DenseNet121 has achieved extremely competitive performance scores (accuracy: 89.23%, precision: 89.69%, recall: 89.23%, and F1-score: 89.11%). Still, our proposed framework demonstrates improved predictive capabilities, achieving slightly higher scores for performance metrics (accuracy: 90.33%, precision: 90.69%, recall: 90.33%, and F1-score: 90.27%). In addition to this performance gain (accuracy: 1.23%, precision: 1%, recall: 1.23%, and F1-score: 1.30%), the proposed LiteFeatNet also exhibits deployment and development computational efficiencies, with a lower inference time (1.32 vs. 1.59 seconds) and fast-paced training (409.35 vs. 416.62 seconds). Furthermore, the proposed framework also offers 30% (approximately) reduced memory footprint compared to DenseNet121. This modest size reduction is extremely important for deploying in real-world clinical settings.*

**5.5 Evaluation metrics**

**Line # 934-938**

*Although DenseNet121 outperformed the proposed model, its inference time (1.60 seconds) is significantly higher than that of LiteFeatNet (1.33 seconds). Thus, despite a minor performance reduction (accuracy: 0.57%, precision: 0.49%, recall: 0.57% and F1-score: 0.54%) compared with DenseNet121, our model still demonstrates deployment level efficiency (memory and time) on this external dataset.*

**7.2 Ablation study**

**Line # 1160-1236**

*We also evaluated the impact of DIFE and PFMR with two top-performing backbone feature extraction architectures: DenseNet121 and MobileNetV2. To conduct these ablation experiments, we evaluated eight model variants, four for each DenseNet121 and MobileNetV2 backbone feature extractor. The suffixes (A, B, C, and D) denote the consistency in ablation model configurations. The architectural specifications used in these ablation experiments and corresponding evaluation metrics for this second set of experiments are shown in Table 12.*

*Table 12. Ablation experiments for other backbone architectures.*

| *Ablation Model* | *DIFE* | *PFMR* | *Accuracy* | *Precision* | *Recall* | *F1-Score* | *Training Time* | *Inference Time* |
| --- | --- | --- | --- | --- | --- | --- | --- | --- |
| *DenseNet121A* | *✓* | *✓* | *89.78* | *90.21* | *89.78* | *89.71* | *401.93* | *1.57* |
| *DenseNet121B* | *✗* | *✗* | *88.40* | *89.25* | *88.40* | *88.33* | *420.59* | *1.46* |
| *DenseNet121C* | *✓* | *✗* | *89.23* | *90.00* | *89.23* | *89.15* | *401.31* | *1.43* |
| *DenseNet121D* | *✗* | *✓* | *89.50* | *89.87* | *89.50* | *89.44* | *413.68* | *1.53* |
| *MobileNetV2A* | *✓* | *✓* | *86.19* | *86.61* | *86.19* | *86.02* | *350.62* | *1.00* |
| *MobileNetV2B* | *✗* | *✗* | *83.98* | *84.58* | *83.98* | *83.72* | *343.02* | *0.98* |
| *MobileNetV2C* | *✓* | *✗* | *85.36* | *86.16* | *85.36* | *85.11* | *345.75* | *0.98* |
| *MobileNetV2D* | *✗* | *✓* | *86.19* | *86.57* | *86.19* | *86.01* | *361.12* | *1.01* |

*Accuracy, Precision, Recall, and F1-Score (%), Training Time, and Inference Time (seconds)*

*To provide a quantitative measure of the effectiveness of the DIFE, PFMR, and LCC modules, we have evaluated the improvement (%) in the performance of various ablation models by using their corresponding transfer learning architectures as baseline references. The percentage improvement/reduction in the performance of NASNetMobile, DenseNet121, and MobileNetV2, are presented in Fig 16, Fig 17, and Fig 18, respectively.*

***Fig 16. Performance improvement with NASNetMobile backbone.***

***Fig 17. Performance improvement with DenseNet121 backbone.***

***Fig 18. Performance improvement with MobileNetV2 backbone.***

*As shown in Fig 16, the LiteFeatNetB improved the accuracy, precision, recall, and F1-score by 0.96%, 0.91%, 0.96%, and 0.93%, respectively, compared with the reference fine-tuned NASNetMobile. Conversely, the same architectural configuration with DenseNet121 and MobileNetV2 backbones reported considerable performance reduction (accuracy: 0.93%, precision: 0.49%, recall: 0.93%, and F1-score: 0.88%) and (accuracy: 1.29%, precision: 1.18%, recall: 1.29%, and F1-score: 1.37%), as shown in Fig 17 and Fig 18, respectively, in comparison to the baseline transfer learning settings. These results effectively highlight the role of the LCC module in the performance improvement of the proposed framework. It is also revealed that integrating the LCC module solely with DenseNet121 and MobileNetV2 backbones results in performance drop, thereby limiting its effectiveness with these architectures, particularly when integrated alone.*

*As shown in Fig 16, the LiteFeatNetC model improved performance (accuracy: 1.59%, precision: 1.70%, recall: 1.59%, and F1-score: 1.57%) in comparison with the reference baseline. Whereas, according to Fig 17, DenseNet121C marginally increased baseline’s precision and F1-score of the by 0.35% and 0.04%, respectively. The MobileNetV2C, as shown in Fig 18, also reported a fractional performance improvement of 0.33% in accuracy, 0.67% in precision, 0.33% in recall, and 0.27% in F1-score, as compared with MobileNetV2. Thus, integration of the DIFE proved more beneficial for LiteFeatNet than the DenseNet121 and MobileNetV2 backbones, which again validates our design decision behind the proposed framework.*

*The model LiteFeatNetD improved the performance metrics of LiteFeatNetC by 2-fold (approximately), as shown in Fig 16. It achieved improvements of 3.18% in accuracy, 3.23% in precision, 3.18% in recall, and 3.20% in F1-score, compared with the NASNetMobile. As shown in Fig 17, DenseNet121D slightly improved all the performance metrics (accuracy: 0.30%, recall: 0.30%; precision: 0.20%; F1-score: 0.37%) compared to the baseline DenseNet121. Whereas, the configuration MobileNetV2D exhibited substantial improvement across all performance evaluation metrics (accuracy, recall, and F1-score: 1.30%; precision: 1.14%), as given in Fig 18, clearly validating the effectiveness of the PFMR module, particularly for lightweight architectures such as NASNetMobile and MobileNetV2.*

*Interestingly, all the DIFE-PFMR-integrated model variants, LiteFeatNetA, DenseNet121A, and MobileNetV2A, dramatically improved the performance of the corresponding baseline architectures. According to Fig 16, the performance of the NASNetMobile baseline was improved by a comprehensive margin (accuracy: 4.14%, precision: 4.60%, recall: 4.14%, and F1-score: 4.14%). Similarly, DenseNet121A also showed moderate improvements in accuracy (0.62%), precision (0.58%), recall (0.62%), and F1-score (0.67%), as shown in Fig 17. MobileNetV2A, though reported only a marginal increase in precision and F1-score compared with MobileNetV2D, demonstrated a substantial improvement of 1.30% in accuracy, 1.19% in precision, 1.30% in recall, and 1.34% in F1-score (as presented in Fig 18) compared with the baseline version. The performance metrics achieved confirm that the effectiveness of integrating DIFE and PFMR module across different backbones was superior when compared with other configurations.*

*Fig 19 compares the inference times of LiteFeatNetA, DenseNet121A, and MobileNetV2A with their counterparts. According to Fig 19, the ablation models MobileNetV2A, DenseNet121A, and LiteFeatNetA reported lower inference times (1.00, 1.57, and 1.32 seconds, respectively) than those of MobileNetV2, DenseNet121, and NASNetMobile (1.05, 1.59, and 1.34 seconds, respectively). This, once again confirms that the DIFE and PFMR synergy empowers transfer learning models to be computationally efficient, thereby enabling deployment-level efficiency in resource-constrained environments.*

***Fig 19. Comparison of inference times.***

*All results with the configuration utilizing both DIFE and PFMR confirm the synergistic effect of DIFE and PFMR for performance improvement across all the baselines, in particular. Moreover, their integration with NASNetMobile backbone demonstrated the effectiveness, efficiency, and motivation behind the specific architectural choices made for designing a lightweight, performance-centric, and computationally efficient LiteFeatNet architecture.*

**Minor Errors:**

We are grateful to the reviewer for highlighting various language issues, spelling mistakes, and formatting errors. To improve the clarity and overall quality of our manuscript, we have updated the manuscript text at locations pointed out by the reviewer. We have also thoroughly read our manuscript once again to identify such issues, if any. All minor language, punctuation, and formatting issues have been corrected to improve the overall quality, clarity, and readability of our manuscript.

| **Error / Comment** | **Response** |
| --- | --- |
| Line 28: "using 1824 images from to three distinct class labels" , delete "to" (should be "from three distinct class labels") | The updated sentence at line # 28 (**updated: line # 26)** now read as:  **Line # 26**  *“using 1824 images from three distinct class labels”* |
| Line 57: "Cost-effective, non-invasive", missing space after comma | The updated sentence at line # 57 **(updated: line # 60-61)** now appears as:  **Line # 60-61**  *“Cost-effective, non-invasive fundus”* |
| Line 95: "cost-effective, scalable", missing space after comma | The updated content in line # 95 **(updated: line # 102)** now appears as:  **Line # 102**  *“cost-effective, scalable”* |
| Line 94: "implementated", should be "implemented" | Amendments made to the sentence structure |
| Line 175: "Theinclusion", missing space (should be "The inclusion") | We have updated line # 175 **(updated: line # 191-192)** as:  **Line # 191-192**  *“The inclusion”* |
| Line 212: "92.40%accuracy", missing space (should be "92.40% accuracy") | The updated content in line # 212 **(updated: line # 234)** reads as follows:  **Line # 234**  *“92.40% accuracy”* |
| Line 358: "information,and fully leverages" , missing space after comma | The necessary correction has been made in the line # 358 **(updated: line # 410)** as follows:  **Line # 410**  *“information, and fully leverages”* |
| Line 521: "Parameters (in numbers), size (in MBs)The LiteFeatNet", missing space or period before "The" | The sentence content “The LiteFeatNet” was actually part of main text appearing right after a **Table 3**. However, the sentence content was accidentally placed right next to the footnote of the table “Parameters (in numbers), size (in MBs)”. We have corrected this issue by clearly separating the main text from the footnote in line # 521 **(updated: line # 568)**. The updated part appears as follows:  **Line # 568-569**  ***“Parameters (in numbers), size (in MBs)***  *The LiteFeatNet”* |
| Line 854: "94.04%)while requiring", missing space before "while" | The updated line # 854 **(updated: line # 953-954)** now appears as:  **Line # 953-954**  *“94.04%) while requiring”* |

We, once again, sincerely thank the reviewers and the editorial team for their constructive input. We believe that the revisions have comprehensively addressed all the concerns and improved the overall quality of the manuscript.

Sincerely,

Usman Rafi

(On behalf of all authors)
Department of Computer Science,

University of Agriculture, Faisalabad, Punjab, Pakistan
usmanrafiuafcs@gmail.com
